# Hypertension Phenotypes and Mortality Risk in the United States of America: A Data‐Driven Cluster Analysis

**DOI:** 10.1155/ijhy/7193567

**Published:** 2025-12-12

**Authors:** Rodrigo M. Carrillo-Larco, Jithin Sam Varghese, Arshed Quyyumi, K. M. Venkat Narayan, Peter W. F. Wilson, Mohammed K. Ali

**Affiliations:** ^1^ Hubert Department of Global Health, Rollins School of Public Health, Emory University, Atlanta, USA, emory.edu; ^2^ Emory Global Diabetes Research Center of Woodruff Health Sciences Center, Emory University, Atlanta, Georgia, USA, emory.edu; ^3^ Division of Cardiology, Department of Medicine, Emory School of Medicine, Emory University, Atlanta, Georgia, USA, emory.edu; ^4^ Department of Family and Preventive Medicine, Emory School of Medicine, Emory University, Atlanta, Georgia, USA, emory.edu

**Keywords:** artificial intelligence, cardiovascular diseases, machine learning, precision medicine, unsupervised learning

## Abstract

**Background:**

Hypertension is a leading, yet modifiable, cause of mortality worldwide. While current treatment guidelines apply uniformly, variation in outcomes suggests unrecognized biological heterogeneity. Existing classifications based solely on systolic and diastolic blood pressure fail to capture this complexity. We identified data‐driven clinical phenotypes of primary hypertension and examined their associations with mortality.

**Methods:**

Pooled analysis of 10 cross‐sectional surveys (NHANES 1999–2020). Data from 4084 adults (≥ 30 years) with newly diagnosed or undiagnosed hypertension were collected. Hypertension was defined by self‐report (in the last 2 years) or those with undiagnosed high systolic or diastolic blood pressure (≥ 140/90 mmHg). Predictors: age, body mass index, systolic blood pressure, diastolic blood pressure, total cholesterol, high‐density lipoprotein cholesterol (HDL‐c), hemoglobin A1c, and estimated glomerular filtration rate (eGFR). We used these variables because they are readily available in primary care settings, enabling clinical translation of these findings. We used k‐means clustering of eight variables to identify phenotypes. Using mortality data linked to the National Death Index, we estimated the risk of all‐cause and cardiovascular mortality.

**Results:**

Four phenotypes: Early‐onset hypertension (EOH), late‐onset hypertension (LOH), glucose‐related hypertension (GRH), and lipid‐related hypertension (LRH). EOH (37.3%) consisted of younger adults with high BMI and diastolic blood pressure, and low systolic blood pressure and HDL‐c. LOH (32.6%) consisted of older adults with low diastolic blood pressure, total cholesterol, and eGFR. GRH (4.5%) consisted of adults with high BMI and HbA1c. LRH (25.6%) consisted of adults with high systolic blood pressure, total cholesterol, and HDL‐c and low BMI and HbA1c. Compared to EOH, mortality was the highest in GRH (all‐cause: 3.45 [1.80–6.61]; cardiovascular: 5.40 [2.18–13.37]), yet not significant for LOH (1.18 [0.74–1.87]; 1.04 [0.49–2.21]) and LRH (1.01 [0.62–1.63]; 0.93 [0.46–1.87]).

**Conclusions:**

This data‐driven cluster analysis identified four phenotypes with different mortality risks in people with newly diagnosed hypertension.

**What Is Known About the Topic?**

• Research in diabetes has shown that individuals with diabetes are not homogenous. Instead, they can be categorized into distinct phenotypes.
• Each phenotype has its own cardiometabolic profile, treatment response, and disease progression. Whether similar phenotypes exist in hypertension has not been explored.

**What This Study Adds?**

• Using population‐based longitudinal data, we identified four hypertension phenotypes, each with a unique cardiometabolic risk profile and mortality risk.
• This study could drive research into replicating these phenotypes across populations, exploring phenotype‐specific treatments, and understanding disease progression.

**What Is New?**

• This study identified four hypertension phenotypes, early‐onset hypertension (EOH), late‐onset hypertension (LOH), glucose‐related hypertension (GRH), and lipid‐related hypertension (LRH), using data‐driven cluster analysis of variables available in primary care settings. These phenotypes reveal heterogeneity within individuals with hypertension and differences between them in risks of all‐cause and cardiovascular mortality.

**What Is Relevant?**

• The identification of distinct hypertension phenotypes based on clinical variables that are commonly available in primary care settings allows for more precise classification of patients with hypertension. This phenotypic differentiation is crucial because it shows that not all patients have the same risk profile, and therefore, may benefit from tailored management strategies.

**Clinical/Pathophysiological Implications**

• The findings underscore the importance of precision medicine in the management of hypertension. By recognizing the unique risk profiles associated with each phenotype, clinicians can tailor interventions more effectively. Patients classified under the GRH phenotype, who have the highest mortality risk, may require intensive glucose management in addition to blood pressure control. Similarly, the identification of phenotypes such as EOH and LOH can help in developing age‐specific hypertension management protocols. Integrating these phenotypic classifications into electronic health records is feasible and could facilitate their use in clinical practice, promoting real‐time identification and tailored treatment of hypertensive patients.

## 1. Introduction

High blood pressure is a leading modifiable risk factor for mortality in the United States, with one in three adults affected [1‐3]. Current treatment guidelines for primary hypertension recommend pharmacological and nonpharmacological interventions offered universally to all people meeting diagnostic criteria [4‐7]. However, the heterogeneity in control and outcomes suggests that the current paradigm does not sufficiently account for potential differences in clinical or biological characteristics (*phenotypes*) of individuals with primary hypertension. Recent literature highlights clinically meaningful differences among individuals with hypertension, including variations in salt sensitivity, arterial stiffness, sympathetic nervous system activation, and metabolic comorbidities [8]. This heterogeneity underscores the need to stratify individuals with hypertension into distinct phenotypes, each characterized by unique risk profiles and, potentially, differential responses to treatment.

Previous efforts to characterize the heterogeneity in hypertension relied only on categories of systolic and diastolic blood pressure, namely, isolated systolic hypertension, isolated diastolic hypertension, or systolic and diastolic hypertension [9‐11]. Similarly, there have been efforts to characterize phenotypes based on the renin–angiotensin system [12, 13]. These previously characterized phenotypes are useful for crude estimation of risk of cardiovascular complications and studying the diagnostic reproducibility of ambulatory blood pressure [9‐11], as well as for treatment response [12, 13], respectively. However, they are inadequate in characterizing the heterogeneity in pathophysiology, risk of cardiovascular complications, and response to interventions for primary hypertension. Identifying phenotypes of primary hypertension can inform treatment selection strategies. As precision medicine evolves, its success depends on the integration of diverse clinical and physiological data above and beyond blood pressure measurements only to fully characterize the patient’s profile [14, 15]. Such integration is essential to enable individualized risk prediction and guide precision hypertension care.

Our objectives were to identify and describe the distributions of clinical phenotypes, also known as risk clusters, of primary hypertension in adults using nationally representative data from the United States. We also studied the risk of all‐cause and cardiovascular mortality for each phenotype.

## 2. Methods

Anonymized data and materials are publicly available at the National Health and Nutrition Examination Surveys (NHANES) repository and can be accessed at https://www.cdc.gov/nchs/nhanes/index.htm.

### 2.1. Data Sources

The NHANES are population‐based, nationally representative sample surveys designed to assess the health and nutritional status of noninstitutionalized adults and children in the United States [16]. The NHANES is conducted in 2‐year cycles and includes a household interview as well as anthropometric, clinical, and biochemical measurements. For this study, we pooled data from 10 rounds of NHANES from 1999–2000 to 2017 March, 2020 prepandemic. We also used the linked mortality files (National Death Index) for NHANES 1999–2018 participants, developed by the National Center for Health Statistics. This study followed the Strengthening the Reporting of Observational Studies in Epidemiology (STROBE) reporting guideline (Checklist on Supplementary Materials pp. 11–15).

### 2.2. Study Population

We restricted our analysis to adults aged 30 years or older who self‐reported being diagnosed with hypertension within the last 2 years or had measured high blood pressure. This age threshold aligns with global analyses of primary hypertension [1], and the 2‐year criterion is consistent with established practices in similar research [17]. Participants were asked the question: “*Were you told you had hypertension, also called high blood pressure?*” (Supplementary Materials pp. 03–05). Duration since hypertension diagnosis in years was estimated from current age and age at diagnosis as self‐reported by the participants who were asked, “*How old were you when you were first told that you had hypertension or high blood pressure?*” (Supplementary Materials pp. 03–05). Among those adults without prior hypertension diagnosis, we included those with high systolic or diastolic blood pressure (≥ 140/90 mmHg^4^). Systolic and diastolic blood pressures were measured three times using standardized procedures and validated instruments across NHANES waves (Supplementary Materials pp. 03–05). We used the average of the last two blood pressure measurements to identify previously undiagnosed hypertension.

We excluded adults under 30 years because hypertension in younger adults is often attributable to a secondary cause. We excluded those diagnosed more than 2 years before the date of the interview because they may have benefited from the long‐term positive effects of antihypertensive medications and/or may have accumulated negative effects of high blood pressure.

We additionally excluded participants who had missing data for key clinical and biochemical variables, namely, body mass index (BMI), systolic/diastolic blood pressure, fasting plasma glucose, estimated glomerular filtration rate (eGFR), total cholesterol, and triglycerides. Compared to the analytic sample, the excluded participants (*n* = 653 out of 4737) were older (63 vs. 59 years) and had higher systolic blood pressure (151 mmHg vs. 145 mmHg), higher HbA1c (6.0% vs. 5.9%), lower total cholesterol (196 mg/dL vs. 206 mg/dL), and lower BMI (28 kg/m^2^ vs. 29 kg/m^2^) (Table S1).

### 2.3. Data Collection and Variable Specification

To define the phenotypes, we selected eight variables based on key risk factors for primary hypertension: chronological age (years), BMI (kg/m^2^), systolic blood pressure (mmHg), diastolic blood pressure (mmHg), total cholesterol (mg/dL), high‐density lipoprotein cholesterol (HDL‐c) (mg/dL), hemoglobin A1c (HbA1c) (%), and eGFR (mL/min/1.73 m^2^). We used these variables because they are readily available in primary care settings, which could enable clinical translation of our findings.

Height and weight were measured by trained technicians following standardized procedures. Measured weight and height were used to compute BMI. Blood samples were withdrawn by trained laboratory personnel (Supplementary Materials pp. 03–05). The enzymatic method and magnesium/dextran sulfate solution were used to quantify total cholesterol and HDL‐c, respectively. Creatinine was quantified using the enzymatic method. We used the MDRD equation to compute the eGFR as 175 × (serum creatinine in mg/dL) ^−1.154^ x (age in years) ^−0.203^, multiplied by 0.742 if women and by 1.212 if African American. Further details about the NHANES procedures and laboratory methods are available online and in Supplementary Methods pp. 03‐05 [16].

We described sociodemographic and clinical characteristics of the phenotypes, including but not limited to race ethnicity, self‐reported history of heart attack, self‐reported history of stroke, and current smoking status, collected using standardized questionnaires. For descriptive purposes, we additionally computed the 10‐year absolute cardiovascular risk with the pooled cohorts equation [18] implemented in [19] among individuals without a history of stroke or myocardial infarction.

The NHANES also included a medication module in which participants reported the medications they were taking, and they had to show the container to the interviewer. For the descriptive analysis, we abstracted information on whether the participant was taking beta‐blockers, calcium channel blockers, thiazides, angiotensin‐converting enzyme inhibitors, or angiotensin II receptor blockers (Table S2).

Follow‐up data on mortality were available from the date of the survey through December 31^st^ 2019. For the mortality analysis, we included two endpoints: all‐cause mortality and cardiovascular mortality (Table S3). Observations in the latest NHANES wave (2017‐March 2020) did not yet have follow‐up mortality data (Table S4 shows the number of observations with and without mortality data) and were therefore excluded from the mortality analysis; however, these observations were included for clustering and phenotype description. Participants excluded from the mortality analysis were younger (57 vs. 59 years), had lower total cholesterol (197.4 vs. 207.4), had higher diastolic blood pressure (83.5 vs. 77.4), and had higher BMI (30.1 vs. 29.0) (Table S5).

### 2.4. Cluster Analysis

We used k‐means clustering to identify phenotypes of primary hypertension. K‐means was chosen because it is the most common algorithm in biomedical literature [17, 20‐28]. For a technical explanation of the k‐means algorithm, please refer to Supplementary Materials pp. 06‐09. Phenotypes were developed separately for men and women to avoid biological differences driving cluster separation [17]. The eight continuous variables were standardized to have a mean of zero and a standard deviation of one. We used dendrograms with Euclidean distance, the elbow plot, the silhouette score, and the Jaccard coefficient, to decide on the optimal number of phenotypes. Additional description of the k‐means cluster analysis, including the rationale for choosing the optimal number of phenotypes, is provided in the Supplementary Materials pp. 06‐09.

K‐means is a computer method used to find groups, also known as phenotypes or clusters, within a dataset [29, 30]. Intuitively, you have a set of patients with information on different variables. K‐means looks for patients who are more similar to each other and places them in the same group. The K refers to the number of groups you want the computer to find. The method works by starting with random group centers, and then moving them around step by step until patients are sorted into the closest groups. The final output is a set of clusters, each with patients who share similar patterns. This helps see patterns we might not notice by eye, such as subgroups of patients who have specific cardiometabolic profiles that differ between groups.

### 2.5. Statistical Analysis

We presented unweighted descriptive summaries of sociodemographic, clinical, and biochemical variables by phenotype. We estimated cause‐specific hazard ratios of phenotypes with all‐cause and cardiovascular mortality using Cox proportional hazards regression. The time scale was the number of months since the examination date. We built two regression models with sequential adjustment for confounding variables. Model 1 included only the outcome and phenotypes adjusting for sex and age. Model 2 included sex, age, race, current smoking status, and self‐reported history of heart attack and stroke.

Proportional hazards assumptions were not violated based on the test of interaction of Schoenfeld residuals with time in crude models [31]. We considered the complex survey design when reporting estimates of association with all‐cause and cardiovascular mortality. Data management and the k‐means were carried out using R 4.3.0. and Python 3.10.11, whereas the regression models were carried out using Stata SE V19.

### 2.6. Sensitivity Analyses

We explored the association of phenotypes with cardiovascular mortality, treating mortality due to other causes as a competing risk and fitting two models as specified for the main analysis. We additionally used 130/80 mmHg as the threshold to define new hypertension cases and verified whether this case definition yielded the same number of phenotypes by inspecting the dendrogram plots, elbow plots, and silhouette scores; we also described the profiles of the phenotypes defined using the 130/80 mmHg threshold as well as the all‐cause and cardiovascular mortality risk. Finally, the Cox proportional hazards models for all‐cause and cardiovascular mortality were adjusted for the covariates included in Models 1 and 2, as well as the eight variables used to derive the phenotype configuration. While this approach is not ideal, since the clustering variables are included in both the definition of the exposure and as potential confounders, it provides a way to explore whether the associations observed between phenotype membership and mortality risk are independent of the individual components.

### 2.7. Ethics

All NHANES participants provided informed consent to participate in the survey procedures. We analyzed publicly available, deidentified data, and considered this study to pose minimal risk. Institutional Review Board approval was not sought.

## 3. Results

In the pooled NHANES dataset including all survey years, there were 128,809 observations. Applying the selection criteria (age ≥ 30 as well as recent or new hypertension), there were 5103 observations left. After excluding missing observations, the analytic sample consisted of 4084 adults, of whom 2158 (53%) were men and 1926 (47%) were women (Supplementary Materials p. 9). The average age of the analytic sample was 59.2 (standard deviation: 14.7) years, and 16.4% were Mexican American, 8.4% were other Hispanic, 44.2% were non‐Hispanic White, and 21.8% were Non‐Hispanic Black. Of the analytic sample, 3.6% and 3.7% had a history of heart attack and stroke, respectively.

**Figure 1:**
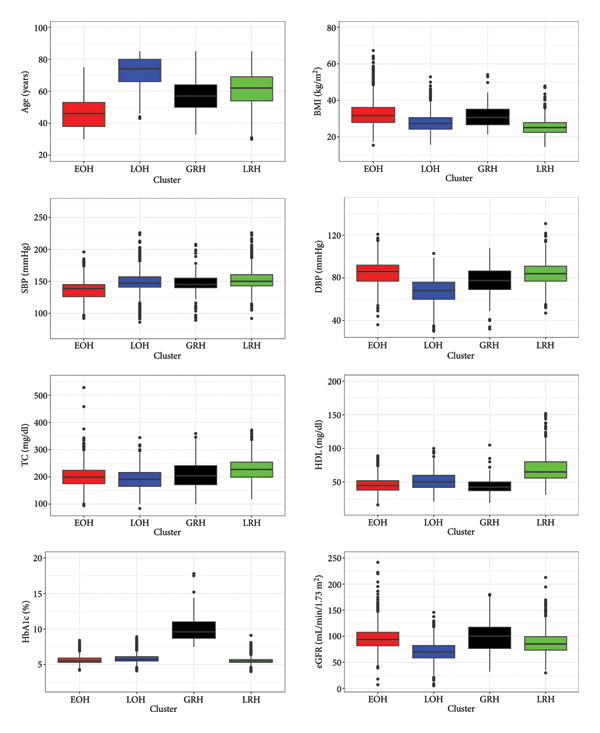
Distribution of the cardiometabolic risk factors by phenotype (cluster). This figure presents boxplots comparing the variables used for the clustering analysis among four distinct clusters labeled as early‐onset hypertension (EOH), late‐onset hypertension (LOH), glucose‐related hypertension (GRH), and lipid‐related hypertension (LRH). Each panel displays the distribution of a specific variable, with the *x*‐axis representing the clusters and the y‐axis showing the variable’s measurement units. The boxplots illustrate the median and interquartile range (IQR), and the whiskers extend to 1.5 times the IQR, with individual points beyond whiskers representing outliers. Top left panel: Age (years) distribution by cluster. This panel shows the variation in participant age across clusters, highlighting differences in central tendency and spread. Top right panel: Body mass index (BMI) in kilograms per square meter (kg/m^2^) by cluster. This panel compares the BMI distributions to assess adiposity differences among the clusters. Second row left panel: Systolic blood pressure (SBP) measured in millimeters of mercury (mmHg) by cluster. This illustrates differences in the upper arterial pressure between clusters. Second row right panel: Diastolic blood pressure (DBP) in mmHg by cluster, indicating variation in lower arterial pressure among the clusters. Third row left panel: total cholesterol (TC) levels in milligrams per deciliter (mg/dL) across clusters, showing the distribution of serum cholesterol concentrations. Third row right panel: High‐density lipoprotein (HDL) cholesterol levels (mg/dL) by cluster, reflecting protective lipid fractions. Bottom left panel: Glycated hemoglobin (HbA1c) percentage by cluster, indicating average blood glucose control over prior months. Bottom right panel: Estimated glomerular filtration rate (eGFR) measured in milliliters per minute per 1.73 square meters (mL/min/1.73 m^2^) by cluster, reflecting kidney function differences. Table S7 shows the ranking of each cardiometabolic risk factor by phenotype. Table S9 shows the mean, median, and standard deviation of the cardiometabolic risk factors by phenotype.

Visual inspection of the eight cardiometabolic risk factors for the four sex‐specific phenotypes (Tables S6 and S7) suggested that the same phenotype labels can be used for men and women (Figure 1; Table S9).

The phenotype entitled EOH consisted of younger adults with high BMI and diastolic blood pressure and low systolic blood pressure and HDL‐c. The phenotype described as LOH consisted of older adults with low diastolic blood pressure, total cholesterol, and eGFR. The phenotype entitled GRH was characterized by adults with high HbA1c and eGFR. The phenotype described as LRH consisted of adults with high systolic blood pressure, total cholesterol, and HDL‐c and low BMI and HbA1c.

EOH (37.3%) was most prevalent in the analytical sample, followed by LOH (32.6%) and LRH (25.6%), and finally GRH (4.5%) (Table 1). In the EOH, LOH, and LRH, most people self‐identified as non‐Hispanic White, whereas in GRH, most people self‐identified as Mexican American. The highest proportion of adults receiving any antihypertensive medication also belonged to phenotype LOH.

**Table 1:** Description of the analytical sample and each phenotype according to the sociodemographic and clinical variables, as well as the eight markers used to derive the phenotypes.

	Pooled analytical sample	Phenotypes			
		Early‐onset hypertension (EOH)	Late‐onset hypertension (LOH)	Glucose‐related hypertension (GRH)	Lipid‐related hypertension (LRH)
	*N* = 4084	*N* = 1525	*N* = 1332	*N* = 182	*N* = 1045
Sex					
Men	52.8%	53.6%	52.6%	64.3%	50.0%
Women	47.2%	46.4%	47.4%	35.7%	50.0%
Race					
Mexican American	16.4%	17.3%	15.0%	33.5%	14.0%
Other Hispanic	8.4%	9.9%	7.7%	13.2%	6.3%
Non‐Hispanic White	44.2%	35.9%	56.5%	22.0%	44.5%
Non‐Hispanic Black	21.8%	26.0%	14.0%	24.2%	25.0%
Others	9.2%	10.9%	6.7%	7.1%	10.2%
Follow‐up time (months)	Median = 113.0	Median = 120.0	Median = 100.4	Median = 100.8	Median = 122.3
Blood pressure < 140 and 90 mmHg , yes	18.5%	28.4%	17.3%	17.6%	5.8%
Blood pressure < 130 and 80 mmHg , yes	11.9%	18.2%	12.2%	11.0%	2.7%
Blood pressure > 180 and 120 mmHg, yes	3.3%	0.2%	4.3%	3.3%	6.4%
History of heart attack, yes	3.6%	1.3%	6.5%	5.5%	2.9%
History of stroke, yes	3.7%	1.6%	6.6%	4.9%	2.7%
Current smoker, yes	20.4%	23.9%	12.7%	22.0%	24.9%
Isolated systolic hypertension, yes	56.0%	30.0%	80.4%	64.3%	61.1%
Isolated diastolic hypertension, yes	10.7%	23.1%	0.5%	6.6%	6.4%
Taking statins	16.8%	8.3%	31.7%	21.4%	9.6%
Taking beta‐blockers	8.3%	4.3%	16.1%	7.1%	4.3%
Taking calcium channel blockers	6.1%	4.1%	11.3%	4.4%	2.7%
Taking thiazides	6.3%	6.9%	8.5%	4.9%	3.0%
Taking angiotensin‐converting‐enzyme inhibitors	10.4%	9.3%	14.9%	18.1%	4.7%
Taking angiotensin II receptor blocker	4.5%	3.6%	7.8%	5.5%	1.2%
All‐cause mortality	23.9%	7.9%	60.8%	5.8%	25.5%
Total person‐years for all‐cause mortality	31,329	11,919	9565	1302	8543
Number of events for all‐cause mortality	796	63	484	46	203
Incidence for all‐cause mortality (per 100 person‐years)	2.54	0.52	5.06	3.53	2.38
Cardiovascular mortality	7.4%	8.1%	61.1%	6.5%	24.3%
Total person‐years for cardiovascular mortality	31,329	11,919	9565	1302	8543
Number of events for cardiovascular mortality	247	20	151	16	60
Incidence for cardiovascular mortality (per 100 person‐years)	0.78	0.16	1.58	1.23	0.70
10‐year absolute cardiovascular risk	Mean = 16.1 SD = 15.7	Mean = 5.7 SD = 5.9	Mean = 28.8 SD = 16.2	Mean = 16.9 SD = 14.2	Mean = 15.4 SD = 13.7
**Clustering variables**					
Current age (expressed in chronological years)	Mean = 59.2 SD = 14.7	Mean = 46.3 SD = 9.5	Mean = 72.5 SD = 8.3	Mean = 56.6 SD = 10.5	Mean = 61.3 SD = 11.3
Body mass index (expressed in kg/m^2^)	Mean = 29.2 SD = 6.5	Mean = 32.7 SD = 6.9	Mean = 27.9 SD = 5.2	Mean = 31.3 SD = 6.0	Mean = 25.4 SD = 4.3
Systolic blood pressure (expressed mmHg)	Mean = 144.7 SD = 18.0	Mean = 136.0 SD = 14.6	Mean = 148.2 SD = 18.2	Mean = 145.2 SD = 19.0	Mean = 152.9 SD = 16.8
Diastolic blood pressure (expressed mmHg)	Mean = 78.5 SD = 13.8	Mean = 84.6 SD = 11.2	Mean = 67.6 SD = 11.7	Mean = 76.8 SD = 12.8	Mean = 84.0 SD = 11.0
Total cholesterol (expressed in mg/dL)	Mean = 205.6 SD = 42.8	Mean = 201.4 SD = 40.4	Mean = 191.3 SD = 37.5	Mean = 208.2 SD = 52.2	Mean = 229.4 SD = 40.9
High‐density lipoprotein (expressed in mg/dL)	Mean = 53.6 SD = 16.9	Mean = 46.1 SD = 11.3	Mean = 51.2 SD = 12.8	Mean = 44.6 SD = 11.8	Mean = 69.0 SD = 19.0
Hemoglobin A1c (expressed in %)	Mean = 5.9 SD = 1.1	Mean = 5.6 SD = 0.6	Mean = 5.9 SD = 0.7	Mean = 10.0 SD = 1.7	Mean = 5.5 SD = 0.5
Estimated glomerular filtration rate (expressed in mL/min/1.73 m^2^)	Mean = 86.0 SD = 24.4	Mean = 96.7 SD = 22.5	Mean = 70.4 SD = 19.1	Mean = 100.1 SD = 29.8	Mean = 87.9 SD = 21.2

Note: Across phenotypes, rows add up to 100%. Isolated systolic hypertension occurs when systolic blood pressure is ≥ 140 mmHg and diastolic blood pressure is < 90 mmHg. Isolated diastolic hypertension when systolic blood pressure was < 140 mmHg and diastolic blood pressure was ≥ 90 mmHg. The 10‐year absolute cardiovascular risk was computed in R with the pooled cohort function and did not include people with history of heart attack or stroke (because the pooled cohorts equation is for primary cardiovascular risk stratification). Sex‐specific results are available in Table S8.

Of the analytic sample, 3327 (81.5%) had information on vital status, among whom 796 died of any cause (all‐cause mortality) and 247 died of cardiovascular‐related causes. The mean duration of follow‐up was 9.4 years (median: 8.8 years). The majority of the analytic sample who died of any cause (60.8%) and cardiovascular causes (61.1%) belonged to phenotype LOH (Table 1).

**Figure 2:**
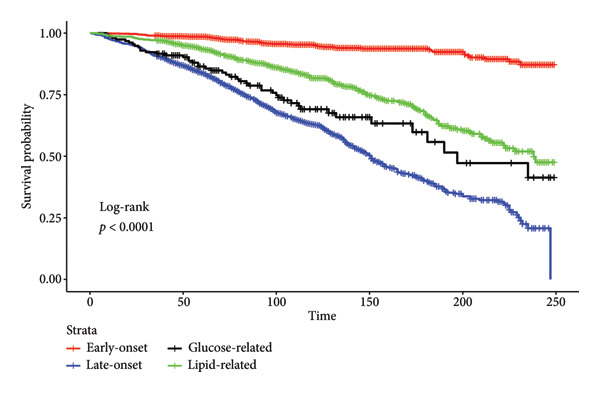
Kaplan–Meier curve for all‐cause mortality. The *p* value is for the log‐rank test. A significant *p* value (< 0.05) suggests there are differences between groups in the probability of the event.

**Figure 3:**
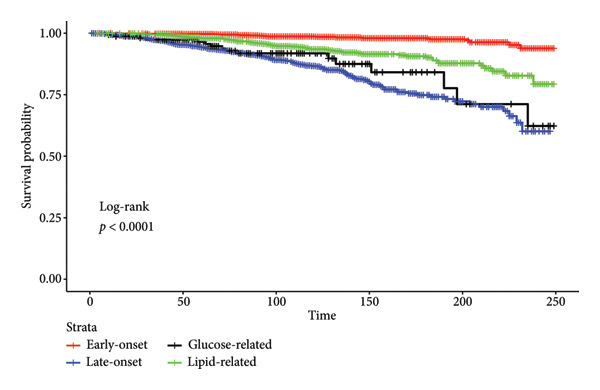
Kaplan–Meier curve for cardiovascular mortality. The *p* value is for the log‐rank test. A significant *p* value (< 0.05) suggests there are differences between groups in the probability of the event.

In both regression models, the GRH phenotype was the only associated factor with a higher risk of all‐cause (HR: 3.45; *p* value < 0.001) and cardiovascular mortality (HR: 5.40; *p* value < 0.001) (Table 2; Figures 2 and 3). LOH was not strongly associated with all‐cause mortality (HR: 1.18; *p* value = 0.467) nor with cardiovascular mortality (HR: 1.04; *p* value = 0.910). LRH was not strongly associated with all‐cause mortality (HR: 1.01; *p* value = 0.953) nor with cardiovascular mortality (HR: 0.93; *p* value = 0.850). The subdistribution hazards model, treating mortality due to other causes as a competing risk, also displayed similar associations by phenotypes (Table S10).

**Table 2:** All‐cause and cardiovascular mortality risk associated with the phenotypes.

	Hazard ratio (95% confidence interval; *p* value)	
	Model 1	Model 2
	Outcome: All‐cause mortality	
	*n* = 3327; events = 796	*n* = 3312; events = 791
Early‐onset hypertension (EOH)	1	1
Late‐onset hypertension (LOH)	1.33 (0.87–2.04; 0.179)	1.18 (0.74–1.87; 0.467)
Glucose‐related hypertension (GRH)	**3.49 (1.83-6.65; < 0.001)**	**3.45 (1.80-6.61; < 0.001)**
Lipid‐related hypertension (LRH)	1.19 (0.76–1.88; 0.430)	1.01 (0.62–1.63; 0.953)
	**Outcome: Cardiovascular mortality**	
	*n* = 3327; events = 247	*n* = 3312; events = 247
Early‐onset hypertension (EOH)	1	1
Late‐onset hypertension (LOH)	1.16 (0.57–2.34; 0.666)	1.04 (0.49–2.21; 0.910)
Glucose‐related hypertension (GRH)	**5.52 (2.34-13.01; < 0.001)**	**5.40 (2.18-13.37; < 0.001)**
Lipid‐related hypertension (LRH)	1.04 (0.53–2.04; 0.892)	0.93 (0.46–1.87; 0.850)

Note: Model 1 reports the association of the outcome and phenotype membership after adjusting for sex and age. Model 2 additionally included race, current smoking status, and self‐reported history of heart attack and stroke. These regression models accounted for the complex survey design of NHANES. Bold values represent the statistically significant results.

In a sensitivity analysis using 130/80 mmHg to define hypertension cases, we identified the same number of phenotypes (Supplementary Materials pp. 06–09). Moreover, the underlying profiles of the phenotypes defined using the 130/80 mmHg threshold were consistent with the main findings (Table S11); similarly, the all‐cause as well as cardiovascular mortality risk estimates were also consistent with the main findings (Table S12), and so were the regression models accounting for competing risk models (Table S13). Finally, when the Cox proportional hazards models for all‐cause and cardiovascular mortality were further adjusted for the eight variables used to derive the phenotypes, the associations between phenotype membership and mortality outcomes were no longer statistically significant (Table S14).

## 4. Discussion

### 4.1. Main Findings

In this data‐driven cluster analysis of adults with recently diagnosed hypertension in the United States, four phenotypes of primary hypertension were evident, characterized by unique underlying clinical profiles and associated with varying risks of all‐cause and cardiovascular mortality. Age and metabolic profiles played a prominent role in defining the phenotypes and relationships to outcomes. Future work should examine whether individualized interventions can optimize blood pressure control and outcomes for each phenotype better than the current one‐size‐fits‐all paradigm. Just as electronic health records today automatically compute and report the 10‐year absolute cardiovascular risk for each patient, in the future, they could also calculate to which phenotype each patient belongs and report this to the clinician. This would be feasible, assuming that the hypertension phenotypes are further validated in independent samples and their association with other health outcomes is verified.

The phenotype hypothesis was previously explored for other related conditions such as diabetes [22, 23, 25, 27], metabolic dysfunction–associated liver disease [26], and obesity [28]. To our knowledge, this is the first effort to use a data‐driven approach to categorize hypertension phenotypes and examine mortality outcomes. Our findings suggest that there is diversity among patients with high blood pressure and their risk of mortality.

The EOH phenotype had the lowest risk of mortality, suggesting nonpharmacological interventions for weight loss may be an important standard of care [32, 33]. In addition, weight loss medications such as *semaglutide* and *tirzepatide* [34, 35], which are rapidly changing the paradigm of obesity treatment, may have important benefits for people with EOH. Although the EOH phenotype was not associated with a higher mortality risk, it is not a desirable condition. Individuals with this phenotype will experience long‐term cumulative exposure to elevated blood pressure from a young age, increasing their risk of cardiovascular events later in life [36].

Phenotype LOH included older adults and those with impaired kidney function [37]. Diastolic blood pressure was lowest in this group, consistent with the life course trajectory of diastolic blood pressure and with the fact that older people have higher arterial stiffness and pulse pressure [38]. The synergism of older age and declining eGFR could explain why phenotype LOH experienced the highest risk of all‐cause and cardiovascular mortality [39, 40]. Phenotype GRH grouped people with metabolic abnormalities as follows: high HbA1c but low HDL‐c. High HbA1c is a known risk factor for all‐cause and cardiovascular mortality [41]. Phenotype LRH included those with the lowest BMI and HbA1c, but the highest systolic blood pressure and the highest total cholesterol. Paradoxically, phenotype LRH also had the highest HDL‐c. Interpretation of the relationship between kidney function and HbA1c should be approached with caution across all phenotypes, as poor kidney function can bias HbA1c measurements [42].

Observational studies and randomized trials for other cardiometabolic diseases such as diabetes report benefits from tailored therapeutic strategies [25, 43]. This hypothesis for hypertension deserves verification with longitudinal observational studies and ideally with trials. Our work provides early evidence of hypertension phenotypes, which require further validation before they can be applied clinically, similar to how cardiovascular risk scores are used for stratification and for initiating antihypertensive or statin therapy [4, 18]. However, while existing risk stratification tools are designed for the general population, our proposed phenotypes are specific to individuals with hypertension. Future research should assess the performance of current risk stratification tools for hypertensive populations and compare them to phenotype‐based approaches to determine which method offers better predictive accuracy.

We used population‐based data to derive the phenotypes, not limiting our participants to narrowly defined inclusion criteria seen in trials and cohort studies. For all predictors and most covariates, we used information objectively measured rather than self‐reported. We restricted the study population to recently and newly diagnosed people with hypertension to limit variation and confounders, and that the phenotyping approach can be used to manage patients with incident hypertension with the goal of using phenotypes for future treatment selection. Finally, we followed reproducible methods to define the number of phenotypes, and we provided all analytical materials for accountability and independent verification. We also chose variables which are available in routine primary care clinical settings, such as blood pressure and anthropometrics, as well as basic blood biomarkers. This facilitates reproducibility and possibly implementation in routine practice in global communities [44].

There are limitations we must acknowledge. First, we analyzed a data source (NHANES) that used consistent methods across survey rounds. However, we did not use the complex survey design in the statistical analyses, suggesting the need for further replicon of our findings in representative cohorts of adults with hypertension. We do not anticipate geography or calendar year to bias our findings, given that associations between phenotypes and mortality were consistent after adjusting for sociodemographic characteristics. Second, the descriptive statistical analyses did not incorporate the complex survey design parameters of NHANES, including sampling weights, primary sampling units (PSUs), and strata. As a result, as purposively intended, the findings should not be interpreted as nationally representative. Point estimates and confidence intervals at the national level may differ from those presented, given the absence of weighting and survey design adjustments. Our primary objective was not to estimate national prevalence or population‐level statistics but rather to identify distinct phenotypes of primary hypertension and characterize them. Nonetheless, regression models did include the complex survey design of NHANES. Third, we did not have an independent dataset for external validation of the phenotypes or to assess long‐term outcomes. We explored analyzing the Atherosclerosis Risk in Communities (ARIC) [45] because of the publicly available cardiovascular cohorts (BioLINCC [45]). ARIC has the largest sample size and longest follow‐up duration. Nonetheless, after applying our selection criteria (recently diagnosed and new patients with hypertension), there were very few observations left. We are not aware of other population‐based big data sources with a large number of people recently diagnosed with hypertension. Fourth, the study population was from one country only. Whether these phenotypes apply to people with hypertension outside the United States remains unknown. The identified phenotypes require further validation before they are used in clinical practice or epidemiological research elsewhere [20]. Fifth, given the design of NHANES, information was available only on fatal cardiovascular events. We could not assess if the phenotypes predict better fatal and nonfatal cardiovascular events in comparison to established risk stratification tools such as the pooled cohorts equation or the new version by Khan et al. [18, 46]. Sixth, we explicitly target new patients with hypertension. Thus, our phenotypes may not be applicable to people who have lived with hypertension for several years. Phenotypes for people with long‐lasting hypertension are also warranted. Although our study did not include genomic and metabolomic data, our approach is easy to replicate in deeply phenotyped cohorts to understand the pathophysiology of hypertension [24]. Seventh, although our study includes a longitudinal component, it was not designed to establish causality. Therefore, the findings should not be interpreted or used to infer causal relationships. Eighth, although a key strength of our study is the incorporation of longitudinal follow‐up for mortality outcomes, the clustering analysis was based on cross‐sectional baseline data. This limited our ability to account for long‐term variability in the phenotype‐defining variables and to capture potential transitions between phenotypes over time. Ninth, in a sensitivity analysis, we adjusted for the variables used to define the phenotypes (the independent variable). This introduces a degree of circularity, as it involves controlling for elements that are intrinsic to the exposure definition, which could attenuate or distort the observed associations. Therefore, we recommend that readers interpret the main analysis as the primary result and view the sensitivity analysis as a robustness check, illustrating that our findings remain consistent even under conservative and potentially overadjusted conditions. Finally, hypertension classification may change over the course of the disease, affecting control definitions and treatment recommendations. This underscores the need to update this and other phenotyping initiatives to reflect current best practices.

## 5. Conclusions

Our data suggest that a comprehensive examination of patients with newly diagnosed primary hypertension can identify latent subpopulations with distinct clinical profiles and disease progression. These patients may benefit from tailored interventions (i.e., personalized care). We identified four unique phenotypes in people with primary hypertension which were differently associated with all‐cause and cardiovascular mortality, advancing the precision intervention agenda for hypertension. In phenotyping, features are included in the original units, so phenotypes can identify subpopulations even when a cardiometabolic risk factor is not above the diagnostic threshold. The main advantage of this phenotyping approach is the potential for future validation and translation of these phenotypes using routine data from integrated electronic health record systems such as PCORnet or Epic Cosmos [47, 48]. Future work in hypertension should aim to examine antihypertensive treatment responses and clinical outcomes in relation to these phenotypes using trials and real‐world data [21].

**Nomenclature**
ARIC: Atherosclerosis Risk in Communities
BMI: Body mass index
eGFR: Estimated glomerular filtration rate
EOH: Early‐onset hypertension
GRH: Glucose‐related hypertension
LOH: Late‐onset hypertension
LRH: Lipid‐related hypertension
NHANES: National Health and Nutrition Examination Surveys
STROBE: Strengthening the Reporting of Observational Studies in Epidemiology

## Ethics Statement

All participants gave written informed consent before participation in NHANES. We were exempt from ethical approval for the analysis of secondary datasets by the Emory University Institutional Review Board.

## Consent

Please see the Ethics Statement.

## Disclosure

All authors approved the submitted version.

## Conflicts of Interest

The authors declare no conflicts of interest.

## Author Contributions

R.M.C.‐L., J.S.V., and M.K.A. designed the study. R.M.C.‐L. conducted the analysis. RMCL and JSV wrote the first draft. R.M.C.‐L., J.S.V., A.Q., K.M.V.N., P.W.F.W., and M.K.A. contributed to reviewing and editing subsequent drafts and provided with critical input to improve the analytical approach, the interpretation of results, the communication of the results, and the scope of the findings and conclusions. R.M.C‐L. and J.S.V. are equally contributed.

## Funding

The authors received no specific funding for this work.

## Supporting Information

Table 1: Comparison of included and excluded observations for the clustering analysis.

Table 2: Antihypertensive drugs included in each family drug.

Table 3: Specific causes of death included in cardiovascular mortality.

Table 4: Absolute number of observations with and without data with regard to the mortality outcome.

Table 5: Comparison of included and excluded observations in the mortality analysis.

Table 6: Original labels (phenotypes or clusters) from the unsupervised models and equivalent nomenclature used in the manuscript.

Table 7: Ranking of the cardiometabolic risk factors (predictors) used in the clustering analyses by cluster or phenotype.

Table 8: Expanded profiles of the phenotypes by sex.

Table 9: Description of the clusters or phenotypes in terms of the eight cardiometabolic risk factors (predictors) used to develop the clusters or phenotypes.

Table 10: Competing risk regressions.

Table 11: Expanded profiles of the phenotypes when using 130/80 mmHg to define new hypertension cases.

Table 12: All‐cause and cardiovascular mortality risk associated with the phenotypes when using 130/80 mmHg to define new hypertension cases.

Table 13: Competing risk regressions when using 130/80 mmHg to define new hypertension cases.

Table 14: All‐cause and cardiovascular mortality risk associated with the phenotypes including confounders in Model 1 and Model 2 as well as the eight variables used to derive the clusters.

## Supporting information


**Supporting Information** Additional supporting information can be found online in the Supporting Information section.

## Data Availability

The data that support the findings of this study are available in NHANES at https://wwwn.cdc.gov/nchs/nhanes/Default.aspx. These data were derived from the following resources available in the public domain: NHANES, https://wwwn.cdc.gov/nchs/nhanes/Default.aspx.
