# Supplementary material for: Hypertension Phenotypes and Mortality Risk in the United States of America: A Data‐Driven Cluster Analysis
Source: Int J Hypertens. 2025 Dec 12;2025:7193567. doi: 10.1155/ijhy/7193567 (PMC12752864; doi:10.1155/ijhy/7193567)
Supplement: Supplementary file 1 — Supporting Information Additional supporting information can be found online in the Supporting Information section. [file IJHY-2025-7193567-s001.doc]

**Hypertension phenotypes and mortality risk in the USA: a data-driven cluster analysis**

# **Expanded Materials and Methods**

## **1.1. Protocol for measuring blood pressure and laboratory methods by NHANES round.**

| **NHANES** | **Protocol for blood pressure measurement** | **Device** | **Questionnaires** | | **Laboratory methods** |
| --- | --- | --- | --- | --- | --- |
| 2017-March 2020 | [link](https://wwwn.cdc.gov/Nchs/Nhanes/2017-2018/P_BPXO.htm) | Sphygmomanometer / Omron HEM–907XL | BPQ020 - Have you/Has SP ever been told by a doctor or other health professional that you/s/he had hypertension, also called high blood pressure? | BPD035 - How old were you/was SP when you were/he/she was first told that you/he/she had hypertension or high blood pressure? | [link](https://wwwn.cdc.gov/nchs/nhanes/continuousnhanes/labmethods.aspx?Cycle=2017-2020) |
| 2015-2016 | [link](https://wwwn.cdc.gov/Nchs/Nhanes/2015-2016/BPX_I.htm) | Sphygmomanometer | BPQ020 - Have you/Has SP ever been told by a doctor or other health professional that you/s/he had hypertension, also called high blood pressure? | BPD035 - How old were you/was SP when you were/he/she was first told that you/he/she had hypertension or high blood pressure? | [link](https://wwwn.cdc.gov/nchs/nhanes/continuousnhanes/labmethods.aspx?BeginYear=2015) |
| 2013-2014 | [link](https://wwwn.cdc.gov/Nchs/Nhanes/2013-2014/BPX_H.htm) | Sphygmomanometer | BPQ020 - Have you/Has SP ever been told by a doctor or other health professional that you/s/he had hypertension, also called high blood pressure? | BPD035 - How old were you/was SP when you were/he/she was first told that you/he/she had hypertension or high blood pressure? | [link](https://wwwn.cdc.gov/nchs/nhanes/continuousnhanes/labmethods.aspx?BeginYear=2013) |
| 2011-2012 | [link](https://wwwn.cdc.gov/Nchs/Nhanes/2011-2012/BPX_G.htm) | Sphygmomanometer | BPQ020 - Have you/Has SP ever been told by a doctor or other health professional that you/s/he had hypertension, also called high blood pressure? | BPD035 - How old were you/was SP when you were/he/she was first told that you/he/she had hypertension or high blood pressure? | [link](https://wwwn.cdc.gov/nchs/nhanes/continuousnhanes/labmethods.aspx?BeginYear=2011) |
| 2009-2010 | [link](https://wwwn.cdc.gov/Nchs/Nhanes/2009-2010/BPX_F.htm) | Sphygmomanometer | BPQ020 - Have you/Has SP ever been told by a doctor or other health professional that you/s/he had hypertension, also called high blood pressure? | BPD035 - How old were you/was SP when you were/he/she was first told that you/he/she had hypertension or high blood pressure? | [link](https://wwwn.cdc.gov/nchs/nhanes/continuousnhanes/labmethods.aspx?BeginYear=2009) |
| 2007-2008 | [link](https://wwwn.cdc.gov/Nchs/Nhanes/2007-2008/BPX_E.htm) | Sphygmomanometer | BPQ020 - Have you/Has SP ever been told by a doctor or other health professional that you/s/he had hypertension, also called high blood pressure? | BPD035 - How old were you/was SP when you were/he/she was first told that you/he/she had hypertension or high blood pressure? | [link](https://wwwn.cdc.gov/nchs/nhanes/continuousnhanes/labmethods.aspx?BeginYear=2007) |
| 2005-2006 | [link](https://wwwn.cdc.gov/Nchs/Nhanes/2005-2006/BPX_D.htm) | Sphygmomanometer | BPQ020 - Have you/Has SP ever been told by a doctor or other health professional that you/s/he had hypertension, also called high blood pressure? | Not available | [link](https://wwwn.cdc.gov/nchs/nhanes/continuousnhanes/labmethods.aspx?BeginYear=2005) |
| 2003-2004 | [link](https://wwwn.cdc.gov/Nchs/Nhanes/2003-2004/BPX_C.htm) | Sphygmomanometer | BPQ020 - Have you/Has SP ever been told by a doctor or other health professional that you/s/he had hypertension, also called high blood pressure? | Not available | [link](https://wwwn.cdc.gov/nchs/nhanes/continuousnhanes/labmethods.aspx?BeginYear=2003) |
| 2001-2002 | [link](https://wwwn.cdc.gov/Nchs/Nhanes/2001-2002/BPX_B.htm) | Sphygmomanometer | BPQ020 - Have you/Has SP ever been told by a doctor or other health professional that you/s/he had hypertension, also called high blood pressure? | Not available | [link](https://wwwn.cdc.gov/nchs/nhanes/continuousnhanes/labmethods.aspx?BeginYear=2001) |
| 1999-2000 | [link](https://wwwn.cdc.gov/Nchs/Nhanes/1999-2000/BPX.htm) | Sphygmomanometer | BPQ020 - Have you/Has SP ever been told by a doctor or other health professional that you/s/he had hypertension, also called high blood pressure? | Not available | [link](https://wwwn.cdc.gov/nchs/nhanes/continuousnhanes/labmethods.aspx?BeginYear=1999) |

We included NHANES 2017-March 2020, instead of NHANES 2017-2018 and NHANES 2019-2020, to include a larger sample size while avoiding overlaps.

## **1.2. Number of clusters**

To select the number of clusters we followed both a data-driven and expert knowledge approach. That is, we computed several metrics to narrow down the potential number of clusters, and we then decided the final number of clusters as informed by experts in cardiometabolic health.

**First**, we drew a dendrogram to explore the number of clusters. We drew dendrograms for men and women; see figures below. For men, the dendrograms suggested the optimal number of clusters was 4 (4 distinct colors or clusters were observed). For women, the dendrograms suggested the optimal number of clusters was 3 (3 distinct colors or clusters were observed). In summary, the dendrograms suggested that the number of clusters would most likely be between 3 and 4 for both men and women.

| **Dendrogram for men** | **Dendrogram for women** |
| --- | --- |
| 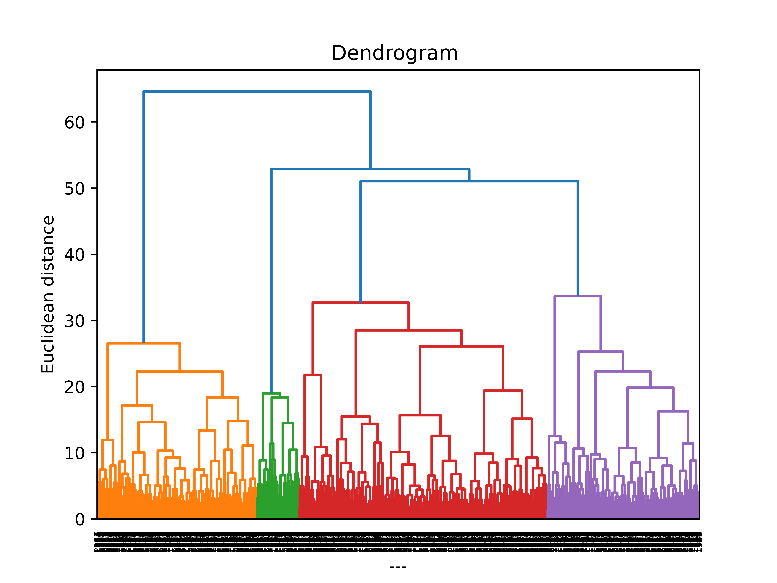 | 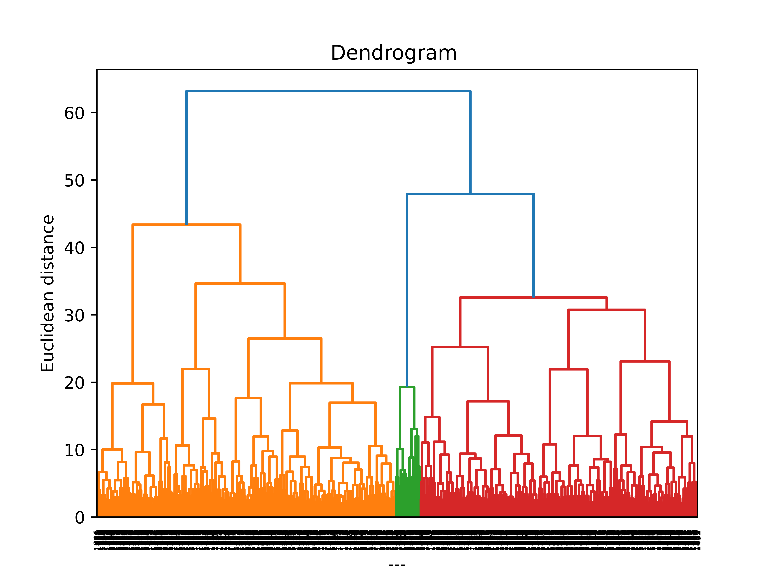 |
| **Using 130/80 mmHg to define new hypertension cases** | |
| 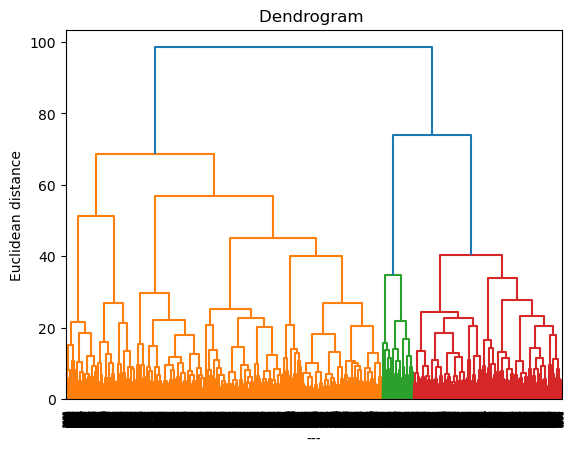 | 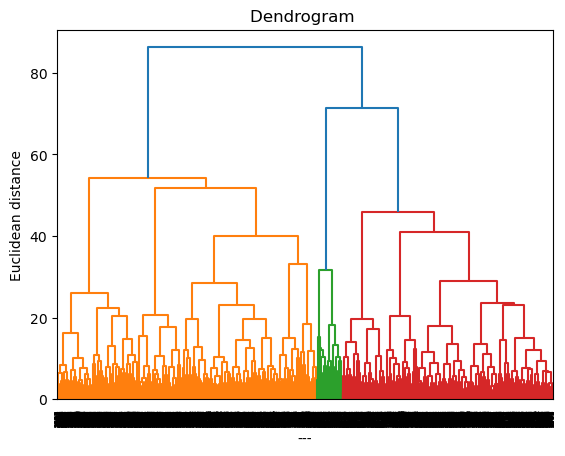 |

**Second**, we plotted the loss (y-axis) as a function of the number of clusters (x-axis). This is commonly referred to as the *Elbow Plot*, because the optimal number of clusters will be at the inflection point (*elbow*). Overall, and consistent with the dendrograms as described above, the optimal number of clusters seemed to be between 3 and 5.

| **Elbow plot for men** | | | **Elbow plot for women** | | |
| --- | --- | --- | --- | --- | --- |
| 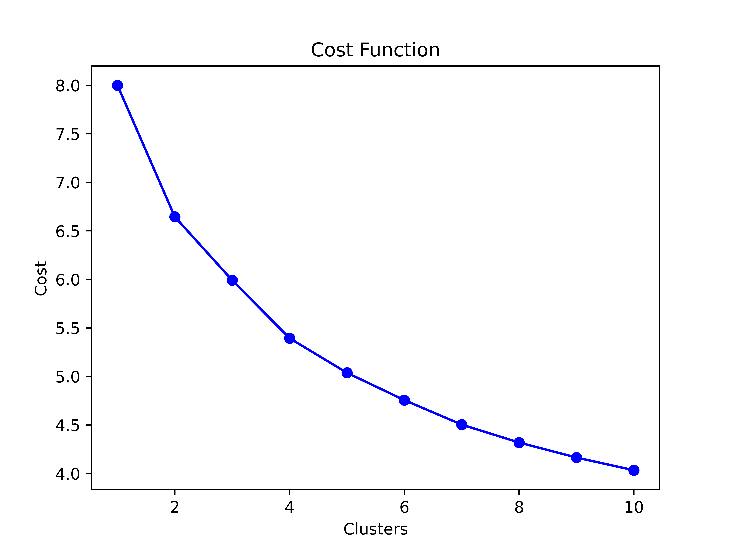 | | | 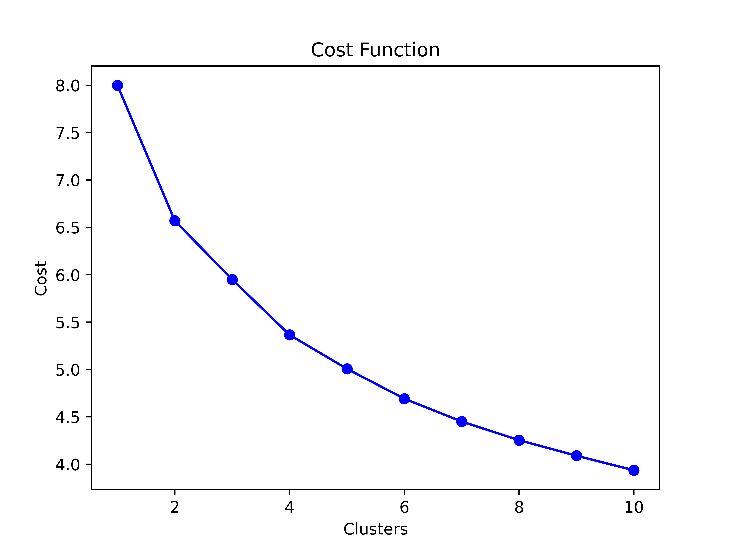 | | |
| **Using 130/80 mmHg to define new hypertension cases** | | | | | |
| **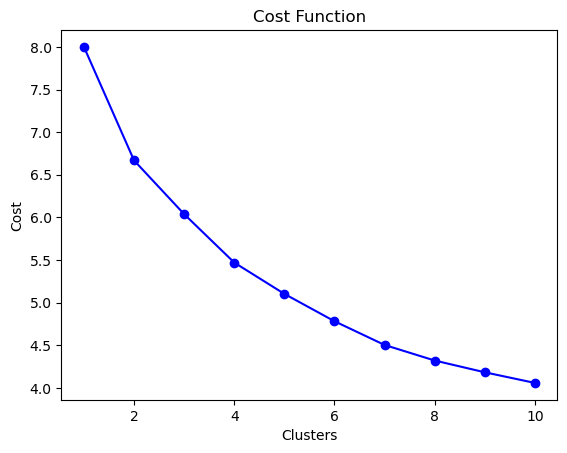** | | | **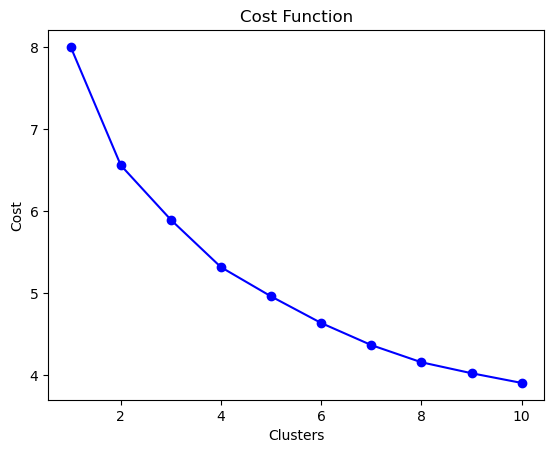** | | |
| **X-axis** | **Y-axis** | **Difference between two consecutive rows in the main analysis** | **X-axis** | **Y-axis** | **Difference between two consecutive rows in the main analysis** |
| 1 | 8.0 |  | 1 | 8.0 |  |
| 2 | 6.6 | 1.4 | 2 | 6.6 | 1.4 |
| 3 | 6.0 | 0.6 | 3 | 6.0 | 0.6 |
| 4 | 5.4 | 0.6 | 4 | 5.4 | 0.6 |
| 5 | 5.0 | 0.4 | 5 | 5.0 | 0.4 |
| 6 | 4.8 | 0.2 | 6 | 4.7 | 0.3 |
| 7 | 4.5 | 0.3 | 7 | 4.5 | 0.2 |
| 8 | 4.3 | 0.2 | 8 | 4.3 | 0.2 |
| 9 | 4.2 | 0.2 | 9 | 4.1 | 0.2 |
| 10 | 4.0 | 0.2 | 10 | 3.9 | 0.2 |

**Third**, we computed the Silhouette score. This metric suggested 4 clusters was the optimal stratification for men and women with a score of 0.16 for both men and women. Notably, the highest Silhouette score was observed for 2 clusters (0.17 in men and 0.18 in women); however, this option was discarded because: i) only 2 clusters will not capture enough variability in people with hypertension; ii) other metrics did not support 2 clusters; and iii) the absolute difference in the Silhouette scores was not substantial (i.e., from 0.17 to 0.16 in men, and from 0.18 to 0.16 in women).

| **Silhouette score – Men //** Using 130/80 mmHg to define new hypertension cases | | **Silhouette score – Women //** Using 130/80 mmHg to define new hypertension cases | |
| --- | --- | --- | --- |
| **Cluster** | **Score** | **Cluster** | **Score** |
| 2 | 0.17 // 0.18 | 2 | 0.18 // 0.18 |
| 3 | 0.15 // 0.19 | 3 | 0.14 // 0.19 |
| 4 | 0.16 // 0.16 | 4 | 0.16 // 0.16 |
| 5 | 0.14 // 0.14 | 5 | 0.15 // 0.13 |
| 6 | 0.14 // 0.13 | 6 | 0.13 // 0.14 |
| 7 | 0.12 // 0.13 | 7 | 0.13 // 0.14 |
| 8 | 0.12 // 0.12 | 8 | 0.13 // 0.14 |
| 9 | 0.12 // 0.11 | 9 | 0.13 // 0.13 |
| 10 | 0.12 // 0.12 | 10 | 0.13 // 0.12 |

**Fourth**, we computed the Jaccard coefficient in R using the *Clusterboot* function^1^. The documentation for this function suggests that the Jaccard similarity mean value should be ≥0.75. We decided to consider clusters where: i) most clusters had a coefficient above 0.75, and ii) no cluster had a coefficient <0.60, because the documentation suggests that clusters with a coefficient <0.60 should not be trusted. The Jaccard coefficients also showed that the optimal number of clusters was 4.

| **Men** | | | | | | | | | |
| --- | --- | --- | --- | --- | --- | --- | --- | --- | --- |
| **Clusters** | **1** | **2** | **3** | **4** | **5** | **6** | **7** | **8** | **9** |
| **Clusterwise Jaccard bootstrap (omitting multiple points) mean:** | 0.93 | 0.94 |  |  |  |  |  |  |  |
|  | 0.86 | 0.88 | 0.74 |  |  |  |  |  |  |
|  | 0.83 | 0.74 | 0.87 | 0.89 |  |  |  |  |  |
|  | 0.73 | 0.88 | 0.69 | 0.82 | 0.60 |  |  |  |  |
|  | 0.74 | 0.92 | 0.58 | 0.70 | 0.3 | 0.55 |  |  |  |
|  | 0.69 | 0.73 | 0.73 | 0.93 | 0.48 | 0.56 | 0.82 |  |  |
|  | 0.59 | 0.75 | 0.58 | 0.40 | 0.92 | 0.44 | 0.81 | 0.47 |  |
|  | 0.51 | 0.77 | 0.66 | 0.92 | 0.38 | 0.52 | 0.78 | 0.50 | 0.31 |
| **Women** | | | | | | | | | |
| **Clusters** | **1** | **2** | **3** | **4** | **5** | **6** | **7** | **8** | **9** |
| **Clusterwise Jaccard bootstrap (omitting multiple points) mean:** | 0.95 | 0.94 |  |  |  |  |  |  |  |
|  | 0.71 | 0.86 | 0.70 |  |  |  |  |  |  |
|  | 0.75 | 0.85 | 0.81 | 0.64 |  |  |  |  |  |
|  | 0.64 | 0.47 | 0.79 | 0.66 | 0.56 |  |  |  |  |
|  | 0.78 | 0.67 | 0.86 | 0.75 | 0.66 | 0.65 |  |  |  |
|  | 0.78 | 0.65 | 0.90 | 0.64 | 0.41 | 0.66 | 0.54 |  |  |
|  | 0.78 | 0.62 | 0.90 | 0.65 | 0.53 | 0.65 | 0.60 | 0.68 |  |
|  | 0.72 | 0.50 | 0.92 | 0.44 | 0.52 | 0.51 | 0.57 | 0.71 | 0.68 |

| **Using 130/80 mmHg to define new hypertension cases** | | | | | | | | | |
| --- | --- | --- | --- | --- | --- | --- | --- | --- | --- |
| **Men** | | | | | | | | | |
| **Clusters** | **1** | **2** | **3** | **4** | **5** | **6** | **7** | **8** | **9** |
| **Clusterwise Jaccard bootstrap (omitting multiple points) mean:** | 0.95 | 0.97 |  |  |  |  |  |  |  |
|  | 0.88 | 0.69 | 0.85 |  |  |  |  |  |  |
|  | 0.93 | 0.83 | 0.85 | 0.90 |  |  |  |  |  |
|  | 0.86 | 0.38 | 0.65 | 0.61 | 0.91 |  |  |  |  |
|  | 0.86 | 0.51 | 0.61 | 0.51 | 0.94 | 0.73 |  |  |  |
|  | 0.89 | 0.77 | 0.86 | 0.85 | 0.87 | 0.89 | 0.96 |  |  |
|  | 0.64 | 0.75 | 0.83 | 0.64 | 0.83 | 0.88 | 0.95 | 0.80 |  |
|  | 0.50 | 0.71 | 0.82 | 0.71 | 0.83 | 0.84 | 0.94 | 0.64 | 0.73 |
| **Women** | | | | | | | | | |
| **Clusters** | **1** | **2** | **3** | **4** | **5** | **6** | **7** | **8** | **9** |
| **Clusterwise Jaccard bootstrap (omitting multiple points) mean:** | 0.95 | 0.96 |  |  |  |  |  |  |  |
|  | 0.85 | 0.85 | 0.71 |  |  |  |  |  |  |
|  | 0.78 | 0.89 | 0.82 | 0.89 |  |  |  |  |  |
|  | 0.91 | 0.72 | 0.60 | 0.79 | 0.57 |  |  |  |  |
|  | 0.58 | 0.78 | 0.70 | 0.94 | 0.73 | 0.79 |  |  |  |
|  | 0.72 | 0.84 | 0.87 | 0.95 | 0.79 | 0.87 | 0.76 |  |  |
|  | 0.93 | 0.79 | 0.87 | 0.84 | 0.73 | 0.83 | 0.83 | 0.85 |  |
|  | 0.94 | 0.79 | 0.65 | 0.72 | 0.42 | 0.80 | 0.67 | 0.78 | 0.49 |

**Finally**, based on these four empirical metrics, and agreed on by the authors based on epidemiological and clinical considerations, we decided to model **4 clusters** of people with hypertension. Selecting the optimal number of clusters is not an exact science and no single metric can unequivocally define the number of clusters. We computed four metrics and incorporated expert knowledge in our decision process, which is transparent as herein reported. The analysis code in Python and R is also available.

## **1.3. Sample selection**

We started with a pooled dataset of all available NHANES waves [n=116,876]

We excluded the 2017-2018 NHANES wave; we restricted the sample to people 30 years and above; and we restricted the sample to people with recent or new hypertension diagnosis [n=4,737]

Finally, we excluded observations with missing data in the features (predictors) used to derive the phenotypes (also known as clusters) [n=4,084]

# **REFERENCES**

# **Supplementary Checklist. STROBE.**

|  | | Item No | Recommendation |
| --- | --- | --- | --- |
| **Title and abstract** | | 1 | (*a*) Indicate the study’s design with a commonly used term in the title or the abstract |
|  |  |  | (*b*) Provide in the abstract an informative and balanced summary of what was done and what was found |
| Introduction | | | |
| Background/rationale | | 2 | Explain the scientific background and rationale for the investigation being reported |
| Objectives | | 3 | State specific objectives, including any prespecified hypotheses |
| Methods | | | |
| Study design | | 4 | Present key elements of study design early in the paper |
| Setting | | 5 | Describe the setting, locations, and relevant dates, including periods of recruitment, exposure, follow-up, and data collection |
| Participants | | 6 | (*a*) *Cohort study*—Give the eligibility criteria, and the sources and methods of selection of participants. Describe methods of follow-up  *Case-control study*—Give the eligibility criteria, and the sources and methods of case ascertainment and control selection. Give the rationale for the choice of cases and controls  *Cross-sectional study*—Give the eligibility criteria, and the sources and methods of selection of participants |
|  |  |  | (*b*) *Cohort study*—For matched studies, give matching criteria and number of exposed and unexposed  *Case-control study*—For matched studies, give matching criteria and the number of controls per case |
| Variables | | 7 | Clearly define all outcomes, exposures, predictors, potential confounders, and effect modifiers. Give diagnostic criteria, if applicable |
| Data sources/ measurement | | 8* | For each variable of interest, give sources of data and details of methods of assessment (measurement). Describe comparability of assessment methods if there is more than one group |
| Bias | | 9 | Describe any efforts to address potential sources of bias |
| Study size | | 10 | Explain how the study size was arrived at |
| Quantitative variables | | 11 | Explain how quantitative variables were handled in the analyses. If applicable, describe which groupings were chosen and why |
| Statistical methods | | 12 | (*a*) Describe all statistical methods, including those used to control for confounding |
|  |  |  | (*b*) Describe any methods used to examine subgroups and interactions |
|  |  |  | (*c*) Explain how missing data were addressed |
|  |  |  | (*d*) *Cohort study*—If applicable, explain how loss to follow-up was addressed  *Case-control study*—If applicable, explain how matching of cases and controls was addressed  *Cross-sectional study*—If applicable, describe analytical methods taking account of sampling strategy |
|  |  |  | (*e*) Describe any sensitivity analyses |
| Results | | | |
| Participants | 13* | (a) Report numbers of individuals at each stage of study—eg numbers potentially eligible, examined for eligibility, confirmed eligible, included in the study, completing follow-up, and analysed | |
|  |  | (b) Give reasons for non-participation at each stage | |
|  |  | (c) Consider use of a flow diagram | |
| Descriptive data | 14* | (a) Give characteristics of study participants (eg demographic, clinical, social) and information on exposures and potential confounders | |
|  |  | (b) Indicate number of participants with missing data for each variable of interest | |
|  |  | (c) *Cohort study*—Summarise follow-up time (eg, average and total amount) | |
| Outcome data | 15* | *Cohort study*—Report numbers of outcome events or summary measures over time | |
|  |  | *Case-control study—*Report numbers in each exposure category, or summary measures of exposure | |
|  |  | *Cross-sectional study—*Report numbers of outcome events or summary measures | |
| Main results | 16 | (*a*) Give unadjusted estimates and, if applicable, confounder-adjusted estimates and their precision (eg, 95% confidence interval). Make clear which confounders were adjusted for and why they were included | |
|  |  | (*b*) Report category boundaries when continuous variables were categorized | |
|  |  | (*c*) If relevant, consider translating estimates of relative risk into absolute risk for a meaningful time period | |
| Other analyses | 17 | Report other analyses done—eg analyses of subgroups and interactions, and sensitivity analyses | |
| Discussion | | | |
| Key results | 18 | Summarise key results with reference to study objectives | |
| Limitations | 19 | Discuss limitations of the study, taking into account sources of potential bias or imprecision. Discuss both direction and magnitude of any potential bias | |
| Interpretation | 20 | Give a cautious overall interpretation of results considering objectives, limitations, multiplicity of analyses, results from similar studies, and other relevant evidence | |
| Generalisability | 21 | Discuss the generalisability (external validity) of the study results | |
| Other information | | | |
| Funding | 22 | Give the source of funding and the role of the funders for the present study and, if applicable, for the original study on which the present article is based | |

*Give information separately for cases and controls in case-control studies and, if applicable, for exposed and unexposed groups in cohort and cross-sectional studies.

**Note:** An Explanation and Elaboration article discusses each checklist item and gives methodological background and published examples of transparent reporting. The STROBE checklist is best used in conjunction with this article (freely available on the Web sites of PLoS Medicine at http://www.plosmedicine.org/, Annals of Internal Medicine at http://www.annals.org/, and Epidemiology at http://www.epidem.com/). Information on the STROBE Initiative is available at www.strobe-statement.org.

# **Table S1. Comparison of included and excluded observations for the clustering analysis.**

| **Variable** | **Included (n=4,084)** | **Excluded (n=653)** | **p-value** |
| --- | --- | --- | --- |
| Age (mean; years) | 59.2 | 63.4 | <0.001 |
| Sex (%) |  |  |  |
| Men | 52.8% | 50.4% | 0.260 |
| Women | 47.2% | 49.6% |
| Systolic blood pressure (mean; mmHg) | 144.7 | 150.6 | <0.001 |
| Diastolic blood pressure (mean; mmHg) | 78.5 | 78.0 | 0.553 |
| HbA1c (mean; %) | 5.9 | 6.0 | 0.031 |
| Total cholesterol (mean; mg/dl) | 205.6 | 195.8 | <0.001 |
| HDL cholesterol (mean; mg/dl) | 53.6 | 53.9 | 0.778 |
| Body mass index (mean; kg/m^2^) | 29.2 | 28.5 | 0.036 |
| eGFR (mean; mL/min/1.73m^2^) | 86.0 | 81.4 | 0.082 |

Comparisons for numeric variables with two-sided unpaired T-tests. Comparisons for categorical data with Chi2 test. P-values <0.05 were considered statistically significant.

# **Table S2. Antihypertensive drugs included in each family drug.**

| **Drug family** | **Examples** |
| --- | --- |
| Beta-blockers | 'atenolol', 'betaxolol', 'bisoprolol', 'metoprolol', 'nadolol', 'propranolol', 'timolol' |
| Calcium channel blockers | 'diltiazem', 'verapamil', 'amlodipine', 'felodipine', 'isradipine', 'nicardipine', 'nifedipine', 'nisoldipine' |
| Thiazides | 'chlorothiazide', 'chlorthalidone', 'hydrochlorothiazide', 'polythiazide', 'indapamide', 'metolazone', 'metolazone' |
| Angiotensin-converting-enzyme inhibitors | 'benazepril', 'captopril', 'enalapril', 'fosinopril', 'lisinopril', 'moexipril', 'perindopril', 'quinapril', 'ramipril', 'trandolapril' |
| Angiotensin II receptor blocker | 'candesartan', 'eprosartan', 'irbesartan', 'losartan', 'olmesartan', 'telmisartan', 'valsartan' |

# **Table S3. Specific causes of death included in cardiovascular mortality.**

Mortality data linked to NHANES provide two key variables: “all-cause mortality” (*mortstat* variable) and cause-specific mortality (*ucod_leading* variable) where they have grouped overreaching categories namely diseases of heart; malignant neoplasms; chronic lower respiratory; accidents (unintentional injuries); cerebrovascular diseases; Alzheimer’s disease; diabetes mellitus; influenza and pneumonia; nephritis, nephrotic syndrome, and nephrosis; and all other causes.

For the mortality analysis, when the outcome was cardiovascular mortality, we combined diseases of heart and cerebrovascular diseases versus all other causes plus alive (i.e., survivor by the end of follow-up). Because the public mortality files do not include individual ICD-10 codes for each cause of death (i.e., they only released all-cause mortality and broad groups of causes described in the previous paragraph), it was not possible to specifically examine some causes of death such as ischemic heart disease or subtypes of stroke. The causes of death included in those two broad groups (diseases of heart and cerebrovascular diseases) are as follow.^13^

| **Diseases of heart** | **Cerebrovascular disease** |
| --- | --- |
| - Acute rheumatic fever and chronic rheumatic disease (I00-I09) - Hypertensive heart disease (I11) - Hypertensive heart and renal disease (I13) - Ischemic heart diseases (I20-I25) - Other heart diseases (I26-I51) | - Cerebrovascular diseases (I60-I69) |

# **Table S4. Absolute number of observations with and without data with regards to the mortality outcome.**

| **NHANES survey** | **Mortality data NOT missing** | | | | **Mortality data missing** | | | |
| --- | --- | --- | --- | --- | --- | --- | --- | --- |
|  | **Cluster (Phenotypes)** | | | | | | | |
|  | **A** | **B** | **C** | **D** | **A** | **B** | **C** | **D** |
| 1999-2000 | 93 | 94 | 18 | 97 |  |  |  |  |
| 2001-2002 | 86 | 115 | 7 | 98 |  |  |  |  |
| 2003-2004 | 54 | 73 | 5 | 73 |  |  |  |  |
| 2005-2006 | 57 | 84 | 9 | 64 |  |  |  |  |
| 2007-2008 | 182 | 169 | 25 | 111 |  |  |  |  |
| 2009-2010 | 195 | 174 | 27 | 113 |  |  |  |  |
| 2011-2012 | 181 | 133 | 22 | 97 |  |  |  | 1 |
| 2013-2014 | 171 | 153 | 16 | 83 | 1 | 1 |  |  |
| 2015-2016 | 172 | 149 | 26 | 102 | 2 |  |  |  |
| 2017-March 2020 |  |  |  |  | 331 | 188 | 27 | 206 |

# **Table S5. Comparison of included and excluded observations in the mortality analysis.**

| **Variable** | **Mortality not missing (n=3,327)** | **Mortality missing (n=757)** | **p-value** |
| --- | --- | --- | --- |
| Age (mean; years) | 59.5 | 57.7 | 0.002 |
| Sex (%) |  |  |  |
| Men | 52.4% | 54.8% | 0.242 |
| Women | 47.6% | 45.2% |
| Systolic blood pressure (mean; mmHg) | 144.8 | 144.1 | 0.257 |
| Diastolic blood pressure (mean; mmHg) | 77.4 | 83.5 | <0.001 |
| HbA1c (mean; %) | 5.9 | 5.9 | 0.246 |
| Total cholesterol (mean; mg/dl) | 207.4 | 197.4 | <0.001 |
| HDL cholesterol (mean; mg/dl) | 53.4 | 54.2 | 0.219 |
| Body mass index (mean; kg/m^2^) | 29.0 | 30.1 | <0.001 |
| eGFR (mean; mL/min/1.73m^2^) | 86.0 | 86.2 | 0.842 |

Comparisons for numeric variables with two-sided unpaired T-tests. Comparisons for categorical data with Chi2 test. P-values <0.05 were considered statistically significant.

# **Table S6. Original labels (phenotypes or clusters) from the unsupervised models and equivalent nomenclature used in the manuscript.**

| **Labels (phenotypes or clusters) as per the unsupervised machine learning model** | | **Nomenclature used in the manuscript*** |
| --- | --- | --- |
| **Men** | **Women** |
| 3 | 1 | **A** |
| 0 | 2 | **B** |
| 1 | 3 | **C** |
| 2 | 0 | **D** |

*For simplicity and to make the manuscript more friendly for the readers, the original phenotypes were labeled consistently for men and women. The selected nomenclature, combining the sex-specific phenotypes, allowed easier description of the phenotypes, and combined phenotypes with similar underlying profiles. For example, phenotype 3 in men and phenotype 1 in women (hence cluster A), both included the youngest people and those with the highest body mass index and diastolic blood pressure (Supplementary Table 6).

# **Table S7. Ranking of the cardiometabolic risk factors (predictors) used in the clustering analysis by cluster or phenotype.**

| **Cluster*** | **Cluster** | **Names** | **Age** | **BMI** | **SBP** | **DBP** | **TC** | **HDL** | **HbA1c** | **eGFR** |
| --- | --- | --- | --- | --- | --- | --- | --- | --- | --- | --- |
|  |  |  | **Men** | | | | | | | |
| 3 | A | Early-onset hypertension (EOH) | 4^th^ (youngest) | 1^st^ (highest) | 4^th^ (lowest) | 1^st^ (highest) | 3^rd^ | 4^th^ (lowest) | 3^rd^ | 2^nd^ |
| 0 | B | Late-onset hypertension (LOH) | 1^st^ (oldest) | 3^rd^ | 3^rd^ | 4^th^ (lowest) | 4^th^ (lowest) | 2^nd^ | 2^nd^ | 4^th^ (lowest) |
| 1 | C | Glucose-related hypertension (GRH) | 3^rd^ | 2^nd^ | 2^nd^ | 3^rd^ | 2^nd^ | 3^rd^ | 1^st^ (highest) | 1^st^ (highest) |
| 2 | D | Lipid-related hypertension (LRH) | 2^nd^ | 4^th^ (lowest) | 1^st^ (highest) | 2^nd^ | 1^st^ (highest) | 1^st^ (highest) | 4^th^ (lowest) | 3^rd^ |
|  |  |  | **Women** | | | | | | | |
| 1 | A | Early-onset hypertension (EOH) | 4^th^ (youngest) | 1^st^ (highest) | 4^th^ (lowest) | 1^st^ (highest) | 4^th^ (lowest) | 3^rd^ | 3^rd^ | 2^nd^ |
| 2 | B | Late-onset hypertension (LOH) | 1^st^ (oldest) | 3^rd^ | 2^nd^ | 4^th^ (lowest) | 3^rd^ | 2^nd^ | 2^nd^ | 4^th^ (lowest) |
| 3 | C | Glucose-related hypertension (GRH) | 3^rd^ | 2^nd^ | 3^rd^ | 3^rd^ | 2^nd^ | 4^th^ (lowest) | 1^st^ (highest) | 1^st^ (highest) |
| 0 | D | Lipid-related hypertension (LRH) | 2^nd^ | 4^th^ (lowest) | 1^st^ (highest) | 2^nd^ | 1^st^ (highest) | 1^st^ (highest) | 4^th^ (lowest) | 3^rd^ |

Age (years); body mass index (BMl; kg/m^2^); systolic blood pressure (SBP; mmHg); diastolic blood pressure (DBP; mmHg); total cholesterol (TC; mg/dl); HDL cholesterol (mg/dl); HbA1c (%); estimated glomerular filtration rate (eGFR; mL/min/1.73m^2^). Raking was based on the mean regardless of statistically significant differences. For example, 1^st^ suggests that such cluster had the highest mean while 4^th^ suggested that such cluster had the lowest mean. NB: for HDL higher is better (i.e., lower HDL is correlated with higher cardiovascular risk), and for eGFR higher is better too (i.e., lower eGFR correlated with chronic kidney disease), for all the other predictors higher is worse health-wise (i.e., higher age, BMI, SBP, DBP, TC and HbA1c are correlated with mortality). Color scheme: red (highest)>yellow>green>blue (lowest). *Original output from the machine learning models.

# **Table S8. Expanded profiles of the phenotypes by sex.**

|  | **Males** | | | | | **Females** | | | | |
| --- | --- | --- | --- | --- | --- | --- | --- | --- | --- | --- |
|  |  | **Phenotype** | | | |  | **Phenotype** | | | |
|  | **Pooled** | **Early-onset hypertension** | **Late-onset hypertension** | **Glucose-related hypertension** | **Lipid-related hypertension** | **Pooled** | **Early-onset hypertension** | **Late-onset hypertension** | **Glucose-related hypertension** | **Lipid-related hypertension** |
| *N* |  | 818 | 700 | 117 | 523 |  | 707 | 632 | 65 | 522 |
| Race (%) |  |  |  |  |  |  |  |  |  |  |
| Mexican American | 16.3 | 39.3 | 28.5 | 11.4 | 20.8 | 16.6 | 39.4 | 31.2 | 6.6 | 22.8 |
| Other Hispanic | 7.8 | 44.6 | 28.0 | 7.1 | 20.2 | 9.1 | 43.2 | 31.8 | 6.8 | 18.2 |
| Non-Hispanic White | 44.2 | 35.8 | 40.6 | 3.0 | 20.5 | 44.2 | 24.1 | 43.0 | 1.3 | 31.6 |
| Non-Hispanic Black | 22.0 | 36.3 | 22.6 | 6.1 | 35.0 | 21.5 | 54.2 | 19.3 | 3.6 | 22.9 |
| Others | 9.8 | 43.1 | 28.0 | 3.3 | 25.6 | 8.5 | 45.7 | 18.3 | 3.7 | 32.3 |
| History of heart attack – Yes (%) | 4.4 | 12.6 | 60.0 | 10.5 | 16.8 | 2.7 | 15.7 | 56.9 | 0.0 | 27.5 |
| History of stroke – Yes (%) | 3.4 | 9.6 | 68.5 | 8.2 | 13.7 | 4.0 | 23.4 | 49.4 | 3.9 | 23.4 |
| Current smoker – Yes (%) | 25.1 | 40.7 | 21.4 | 5.4 | 32.5 | 15.2 | 49.5 | 18.1 | 3.8 | 28.7 |
| Isolated systolic hypertension | 52.2 | 19.4 | 49.3 | 6.7 | 24.5 | 60.1 | 20.6 | 44.5 | 3.5 | 31.3 |
| Isolated diastolic hypertension | 14.6 | 80.4 | 1.9 | 3.5 | 14.2 | 6.3 | 81.0 | 0.0 | 0.8 | 18.2 |
| Taking beta-blockers (%) | 7.6 | 16.0 | 70.6 | 4.3 | 9.2 | 9.1 | 22.7 | 56.8 | 3.4 | 17.0 |
| Taking calcium channel blockers (%) | 5.7 | 20.2 | 64.5 | 3.2 | 12.1 | 6.5 | 30.2 | 56.3 | 3.2 | 10.3 |
| Taking thiazides (%) | 4.7 | 38.2 | 45.1 | 3.9 | 12.7 | 8.1 | 42.3 | 42.9 | 3.2 | 11.5 |
| Taking Angiotensin-converting-enzyme inhibitors | 10.6 | 29.8 | 49.1 | 8.8 | 12.3 | 10.1 | 37.9 | 44.6 | 6.7 | 10.8 |
| All-cause mortality (%) | 24.4 | 8.9 | 62.0 | 7.3 | 21.8 | 23.4 | 6.8 | 59.5 | 4.1 | 29.7 |
| Cardiovascular mortality (%) | 7.5 | 10.7 | 61.1 | 9.2 | 19.1 | 7.3 | 5.2 | 61.2 | 3.4 | 30.2 |
| **Clustering variables** |  |  |  |  |  |  |  |  |  |  |
| Current age (years) | 57.6 (14.8) | 45 (9.3) | 71.8 (8.5) | 56.9 (11.2) | 58.3 (11.4) | 61.0 (14.3) | 47.9 (9.6) | 73.3 (8) | 56.1 (8.9) | 64.3 (10.3) |
| Body mass index (kg/m^2^) | 28.8 (5.9) | 32.1 (6.2) | 27.2 (4.2) | 31.2 (6.3) | 25.2 (3.7) | 29.6 (7.1) | 33.3 (7.7) | 28.6 (5.9) | 31.4 (5.6) | 25.6 (4.8) |
| Systolic Blood Pressure (mm Hg) | 144.0 (16.6) | 136.1 (13.4) | 146.5 (16.7) | 147 (16.8) | 152.1 (15.8) | 145.5 (19.5) | 135.8 (15.9) | 150.2 (19.5) | 142 (22.1) | 153.6 (17.8) |
| Diastolic Blood Pressure (mm Hg) | 80.8 (13.6) | 86.7 (10.7) | 69.8 (11.6) | 79.4 (12) | 86.3 (11.1) | 76.0 (13.6) | 82.1 (11.4) | 65.1 (11.2) | 72.3 (13) | 81.6 (10.4) |
| Total cholesterol (mg/dL) | 200.9 (43.6) | 204.6 (43.8) | 182.2 (36) | 205.3 (54) | 219.1 (40.3) | 210.8 (41.4) | 197.6 (35.7) | 201.5 (36.5) | 213.2 (49) | 239.8 (38.8) |
| High density lipoprotein (mg/dL) | 49.2 (15.5) | 41.8 (8.7) | 47 (11.4) | 43.1 (10.1) | 64.9 (18.3) | 58.5 (17.1) | 51 (11.9) | 55.8 (12.6) | 47.3 (14) | 73.2 (18.8) |
| Hemoglobin A1c (%) | 5.9 (1.1) | 5.6 (0.6) | 5.9 (0.7) | 9.7 (1.5) | 5.6 (0.5) | 5.9 (1.1) | 5.7 (0.6) | 5.9 (0.7) | 10.5 (2.1) | 5.5 (0.4) |
| Estimated Glomerular Filtration Rate (mL/min/1.73m^2^) | 86.3 (22.7) | 93.7 (20.3) | 71.9 (18.9) | 94.9 (28.2) | 91.8 (20.1) | 85.7 (26.2) | 100.1 (24.4) | 68.7 (19.2) | 109.4 (30.4) | 83.9 (21.5) |

Results are presented as percentages and mean (standard deviation) for categorical and continuous variables, respectively. Across phenotypes rows add up to 100%. Isolated systolic hypertension when systolic blood pressure was ≥140 mmHg and diastolic blood pressure was <90 mmHg. Isolated diastolic hypertension when systolic blood pressure was <140 mmHg and diastolic blood pressure was ≥90 mmHg.

# **Table S9. Description of the clusters or phenotypes in terms of the eight cardiometabolic risk factors (predictors) used to develop the clusters or phenotypes.**

| **Sex** | **Predictor** | **Cluster** | **Mean** | **Median** | **Standard deviation** | **Minimum** | **Maximum** |
| --- | --- | --- | --- | --- | --- | --- | --- |
| Men | Age | A | 45.02 | 45.00 | 9.31 | 30.00 | 75.00 |
| Men | Body Mass Index | A | 32.12 | 31.11 | 6.21 | 18.93 | 63.91 |
| Men | Systolic Blood Pressure | A | 136.14 | 138.00 | 13.37 | 92.00 | 196.00 |
| Men | Diastolic Blood Pressure | A | 86.74 | 90.00 | 10.65 | 44.00 | 121.00 |
| Men | Total Cholesterol | A | 204.64 | 201.00 | 43.77 | 100.00 | 528.00 |
| Men | HDL Cholesterol | A | 41.84 | 41.00 | 8.68 | 16.00 | 77.00 |
| Men | HbA1c | A | 5.60 | 5.50 | 0.55 | 4.20 | 7.90 |
| Men | eGFR MDRD | A | 93.70 | 91.64 | 20.33 | 6.97 | 221.73 |
| Men | Age | B | 71.76 | 73.00 | 8.51 | 44.00 | 85.00 |
| Men | Body Mass Index | B | 27.16 | 26.87 | 4.21 | 15.75 | 44.05 |
| Men | Systolic Blood Pressure | B | 146.48 | 146.00 | 16.65 | 86.00 | 213.00 |
| Men | Diastolic Blood Pressure | B | 69.83 | 71.00 | 11.62 | 34.00 | 103.00 |
| Men | Total Cholesterol | B | 182.16 | 181.00 | 36.03 | 84.00 | 317.00 |
| Men | HDL Cholesterol | B | 47.01 | 45.50 | 11.42 | 21.00 | 100.00 |
| Men | HbA1c | B | 5.85 | 5.70 | 0.67 | 4.10 | 8.90 |
| Men | eGFR MDRD | B | 71.94 | 71.75 | 18.93 | 5.05 | 145.68 |
| Men | Age | C | 56.86 | 57.00 | 11.23 | 33.00 | 85.00 |
| Men | Body Mass Index | C | 31.23 | 29.91 | 6.27 | 21.51 | 54.06 |
| Men | Systolic Blood Pressure | C | 147.00 | 145.00 | 16.81 | 93.00 | 208.00 |
| Men | Diastolic Blood Pressure | C | 79.35 | 80.00 | 11.96 | 34.00 | 108.00 |
| Men | Total Cholesterol | C | 205.32 | 201.00 | 53.95 | 100.00 | 346.00 |
| Men | HDL Cholesterol | C | 43.13 | 41.00 | 10.13 | 26.00 | 85.00 |
| Men | HbA1c | C | 9.68 | 9.30 | 1.46 | 7.50 | 15.20 |
| Men | eGFR MDRD | C | 94.86 | 94.51 | 28.20 | 33.61 | 180.16 |
| Men | Age | D | 58.33 | 60.00 | 11.39 | 30.00 | 85.00 |
| Men | Body Mass Index | D | 25.19 | 25.12 | 3.71 | 15.12 | 36.10 |
| Men | Systolic Blood Pressure | D | 152.14 | 149.50 | 15.75 | 107.00 | 222.00 |
| Men | Diastolic Blood Pressure | D | 86.34 | 88.00 | 11.07 | 52.00 | 122.00 |
| Men | Total Cholesterol | D | 219.11 | 216.00 | 40.28 | 118.00 | 372.00 |
| Men | HDL Cholesterol | D | 64.89 | 62.00 | 18.33 | 33.00 | 150.00 |
| Men | HbA1c | D | 5.55 | 5.50 | 0.47 | 4.10 | 7.60 |
| Men | eGFR MDRD | D | 91.84 | 89.90 | 20.14 | 51.90 | 212.67 |
| Women | Age | A | 47.85 | 47.00 | 9.55 | 30.00 | 74.00 |
| Women | Body Mass Index | A | 33.32 | 32.29 | 7.66 | 15.47 | 67.29 |
| Women | Systolic Blood Pressure | A | 135.78 | 140.00 | 15.88 | 95.00 | 185.00 |
| Women | Diastolic Blood Pressure | A | 82.05 | 83.00 | 11.38 | 36.00 | 117.50 |
| Women | Total Cholesterol | A | 197.58 | 197.00 | 35.72 | 94.00 | 337.00 |
| Women | HDL Cholesterol | A | 51.00 | 50.00 | 11.94 | 21.00 | 89.00 |
| Women | HbA1c | A | 5.66 | 5.60 | 0.59 | 4.20 | 8.40 |
| Women | eGFR MDRD | A | 100.05 | 96.48 | 24.40 | 17.86 | 241.67 |
| Women | Age | B | 73.34 | 75.00 | 7.98 | 43.00 | 85.00 |
| Women | Body Mass Index | B | 28.61 | 27.80 | 5.94 | 16.18 | 52.87 |
| Women | Systolic Blood Pressure | B | 150.17 | 149.00 | 19.51 | 91.00 | 226.00 |
| Women | Diastolic Blood Pressure | B | 65.09 | 66.00 | 11.22 | 30.00 | 95.00 |
| Women | Total Cholesterol | B | 201.50 | 201.00 | 36.53 | 101.00 | 344.00 |
| Women | HDL Cholesterol | B | 55.78 | 55.00 | 12.60 | 25.00 | 96.00 |
| Women | HbA1c | B | 5.92 | 5.80 | 0.70 | 4.30 | 8.90 |
| Women | eGFR MDRD | B | 68.71 | 68.27 | 19.19 | 5.70 | 122.77 |
| Women | Age | C | 56.09 | 57.00 | 8.92 | 34.00 | 75.00 |
| Women | Body Mass Index | C | 31.42 | 31.42 | 5.56 | 21.53 | 42.39 |
| Women | Systolic Blood Pressure | C | 141.98 | 144.00 | 22.10 | 89.00 | 206.00 |
| Women | Diastolic Blood Pressure | C | 72.26 | 71.00 | 12.98 | 32.00 | 97.00 |
| Women | Total Cholesterol | C | 213.23 | 208.00 | 48.97 | 130.00 | 359.00 |
| Women | HDL Cholesterol | C | 47.32 | 45.00 | 13.95 | 19.00 | 105.00 |
| Women | HbA1c | C | 10.46 | 10.30 | 2.07 | 7.90 | 17.80 |
| Women | eGFR MDRD | C | 109.40 | 108.19 | 30.41 | 32.29 | 178.54 |
| Women | Age | D | 64.32 | 64.00 | 10.30 | 36.00 | 85.00 |
| Women | Body Mass Index | D | 25.63 | 25.16 | 4.78 | 14.54 | 47.88 |
| Women | Systolic Blood Pressure | D | 153.61 | 150.00 | 17.84 | 92.00 | 226.00 |
| Women | Diastolic Blood Pressure | D | 81.62 | 82.00 | 10.44 | 47.00 | 131.00 |
| Women | Total Cholesterol | D | 239.78 | 237.00 | 38.80 | 150.00 | 370.00 |
| Women | HDL Cholesterol | D | 73.19 | 71.00 | 18.83 | 31.00 | 152.00 |
| Women | HbA1c | D | 5.52 | 5.50 | 0.44 | 4.00 | 9.10 |
| Women | eGFR MDRD | D | 83.90 | 81.86 | 21.47 | 29.77 | 194.50 |

# **Table S10. Competing risk regressions.**

|  | **Hazard Ratio (95% Confidence Interval)** | |
| --- | --- | --- |
|  | **Model 1** | **Model 2** |
|  | **1=censored (survived; n=2,531), 2=cardiovascular mortality (n=247); 3=other causes (n=549)** | |
| Early-onset hypertension (EOH) | 1 | 1 |
| Late-onset hypertension (LOH) | 0.78 (0.44-1.40) | 0.71 (0.39-1.28) |
| Glucose-related hypertension (GRH) | **2.10 (1.02-4.31)** | 1.96 (0.91-4.24) |
| Lipid-related hypertension (LRH) | 0.85 (0.49-1.48) | 0.77 (0.44-1.34) |

Competing risk models with *tidycmprsk* in R. Model 1 reports the association of the outcome and phenotype membership after adjusting for sex and age. Model 2 additionally included race, current smoking status, and self-reported history of heart attack and stroke. These regressions do not include the complex survey design of NHANES because the model does not allow this specification.

# **Table S11. Expanded profiles of the phenotypes when using 130/80 mmHg to define new hypertension cases.**

|  | **Pooled analytical sample** | **Phenotypes** | | | |
| --- | --- | --- | --- | --- | --- |
|  |  | **Early-onset hypertension (EOH)** | **Late-onset hypertension (LOH)** | **Glucose-related hypertension (GRH)** | **Lipid-related hypertension (LRH)** |
| *N* | 8,554 | 3,678 | 2,456 | 360 | 2,060 |
| Sex (%) |  |  |  |  |  |
| Men | 56.5 | 58.7 | 55.3 | 65.8 | 52.5 |
| Women | 43.5 | 41.3 | 44.7 | 34.2 | 47.5 |
| Race (%) |  |  |  |  |  |
| Mexican American | 16.9 | 19.4 | 15.0 | 33.6 | 11.8 |
| Other Hispanic | 7.9 | 8.8 | 7.9 | 10.8 | 6.1 |
| Non-Hispanic White | 44.9 | 40.3 | 56.5 | 24.4 | 43.2 |
| Non-Hispanic Black | 20.2 | 21.4 | 14.0 | 21.9 | 25.1 |
| Others | 10.0 | 10.2 | 6.7 | 9.2 | 13.7 |
| Follow-up time (months; median) | 120.9 | 129.6 | 104.9 | 108.7 | 127.3 |
| Blood pressure <140 & 90 mmHg – Yes (%) | 61.1 | 75.0 | 44.2 | 58.6 | 56.9 |
| Blood pressure <130 & 80 mmHg – Yes (%) | 5.7 | 7.3 | 6.0 | 5.8 | 2.5 |
| Blood pressure >180 & 120 mmHg – Yes (%) | 1.6 | 0.0 | 3.9 | 1.7 | 1.4 |
| History of heart attack – Yes (%) | 2.9 | 1.2 | 6.1 | 4.2 | 1.9 |
| History of stroke – Yes (%) | 2.7 | 0.9 | 5.6 | 4.2 | 2.1 |
| Current smoker – Yes (%) | 20.6 | 22.3 | 12.7 | 20.8 | 27.2 |
| Isolated systolic hypertension – Yes (%) | 26.7 | 9.3 | 53.2 | 31.7 | 25.3 |
| Isolated diastolic hypertension – Yes (%) | 5.1 | 8.7 | 0.2 | 3,9 | 4.8 |
| Taking statins (%) | 13.7 | 6.2 | 28.5 | 23.9 | 7.8 |
| Taking beta-blockers (%) | 5.7 | 2.5 | 13.3 | 5.3 | 2.5 |
| Taking calcium channel blockers (%) | 3.7 | 1.8 | 8.1 | 3.3 | 1.7 |
| Taking thiazides (%) | 3.9 | 3.2 | 6.7 | 3.3 | 1.8 |
| Taking angiotensin-converting-enzyme inhibitors (%) | 6.2 | 4.1 | 10.9 | 15.3 | 2.9 |
| Taking angiotensin II receptor blocker (%) | 2.7 | 1.5 | 5.7 | 3.9 | 0.8 |
| All-cause mortality (%) | 17.9 | 11.9 | 64.8 | 5.1 | 18.2 |
| Total person-years for all-cause mortality | 71,127 | 32,394 | 18,309 | 2,618 | 17,807 |
| Number of events for all-cause mortality | 1,262 | 150 | 828 | 64 | 230 |
| Incidence for all-cause mortality (100 person-years) | 1.77 | 0.46 | 4.47 | 2.44 | 1.29 |
| Cardiovascular mortality (%) | 5.1 | 8.9 | 68.4 | 6.7 | 15.9 |
| Total person-years for cardiovascular mortality | 71,127 | 32,394 | 18,309 | 2,618 | 17,807 |
| Number of events for cardiovascular mortality | 358 | 32 | 245 | 24 | 57 |
| Incidence for cardiovascular mortality (100 person-years) | 0.50 | 0.09 | 1.34 | 0.92 | 0.32 |
| 10-year absolute cardiovascular risk (%; (SD)) | 11.4 (13.3) | 4.3 (4.9) | 25.5 (15.3) | 14.7 (13.8) | 8.0 (8.4) |
| **Clustering variables** |  |  |  |  |  |
| Current age (years) | 55.3 (14.6) | 44.9 (9.2) | 71.4 (8.4) | 54.7 (11.2) | 54.8 (11.2) |
| Body mass index (kg/m^2^) | 29.1 (6.4) | 32.1 (6.7) | 27.8 (5.1) | 31.5 (6.5) | 25.1 (4.1) |
| Systolic Blood Pressure (mm Hg) | 136.0 (16.0) | 128.8 (11.9) | 144.9 (16.9) | 137.1 (16.3) | 137.9 (15.4) |
| Diastolic Blood Pressure (mm Hg) | 78.4 (11.4) | 82.7 (8.3) | 68.9 (11.4) | 76.6 (11.6) | 82.2 (9.1) |
| Total cholesterol (mg/dL) | 205.2 (42.2) | 201.3 (39.9) | 195.3 (39.3) | 207.3 (54.1) | 223.8 (41.4) |
| High density lipoprotein (mg/dL) | 52.8 (16.7) | 45.1 (10.6) | 51.7 (13.5) | 43.8 (10.8) | 69.5 (17.9) |
| Hemoglobin A1c (%) | 5.8 (1.1) | 5.6 (0.5) | 5.9 (0.7) | 10.0 (1.8) | 5.5 (0.4) |
| Estimated Glomerular Filtration Rate (mL/min/1.73m^2^) | 88.4 (23.7) | 94.8 (21.4) | 72.6 (18.9) | 104.4 (28.9) | 92.8 (22.4) |

# **Table S12. All-cause and cardiovascular mortality risk associated with the phenotypes when using 130/80 mmHg to define new hypertension cases.**

|  | **Hazard Ratio (95% Confidence Interval; p-value)** | |
| --- | --- | --- |
|  | **Model 1** | **Model 2** |
|  | **Outcome: all-cause mortality** | |
|  | n = 7,060; events = 1,262 | n = 7,060; events = 1,252 |
| Early-onset hypertension (EOH) | 1 | 1 |
| Late-onset hypertension (LOH) | 1.32 (0.94-1.84; 0.100) | 1.20 (0.86-1.68; 0.270) |
| Glucose-related hypertension (GRH) | **2.43 (1.57-3.77; <0.001)** | **2.51 (1.63-3.87; <0.001)** |
| Lipid-related hypertension (LRH) | 1.25 (0.92-1.70; 0.138) | 1.10 (0.82-1.48; 0.498) |
|  | **Outcome: cardiovascular mortality** | |
|  | n = 7,060; events = 358 | n = 7,037; events = 357 |
| Early-onset hypertension (EOH) | 1 | 1 |
| Late-onset hypertension (LOH) | 1.61 (0.84-3.09; 0.147) | 1.44 (0.75-2.76; 0.261) |
| Glucose-related hypertension (GRH) | **4.90 (2.24-10.71; <0.001)** | **4.97 (2.27-10.90; <0.001)** |
| Lipid-related hypertension (LRH) | 1.52 (0.73-3.15; 0.251) | 1.36 (0.67-2.78; 0.385) |

These regressions account for the complex survey design of NHANES.

# **Table S13. Competing risk regressions when using 130/80 mmHg to define new hypertension cases.**

|  | **Hazard Ratio (95% Confidence Interval)** | |
| --- | --- | --- |
|  | **Model 1** | **Model 2** |
|  | **1=censored (survived; n=5,798), 2=cardiovascular mortality (n=358); 3=other causes (n=904)** | |
| Early-onset hypertension (EOH) | 1 | 1 |
| Late-onset hypertension (LOH) | 1.25 (0.80-1.96) | 1.15 (0.73-1.81) |
| Glucose-related hypertension (GRH) | **3.20 (1.84-5.59)** | **3.01 (1.68-5.41)** |
| Lipid-related hypertension (LRH) | 1.25 (0.80-1.95) | 1.12 (0.71-1.77) |

Competing risk models with *tidycmprsk* in R. Model 1 reports the association of the outcome and phenotype membership after adjusting for sex and age. Model 2 additionally included race, current smoking status, and self-reported history of heart attack and stroke. These regressions do not include the complex survey design of NHANES because the model does not allow this specification.

# **Table S14. All-cause and cardiovascular mortality risk associated with the phenotypes including confounders in Model 1 and Model 2 (Table 2 in manuscript) as well as the eight variables used to derive the clusters.**

|  | **Hazard Ratio (95% Confidence Interval; p-value)** | | |
| --- | --- | --- | --- |
|  | **Model 1** | **Model 2** | **Including the eight features used to derive the clusters/phenotypes** |
|  | **Outcome: all-cause mortality** | | |
|  | n = 3,327; events = 796 | n = 3,312; events = 791 | n = 3,312; events = 791 |
| Early-onset hypertension (EOH) | 1 | 1 | 1 |
| Late-onset hypertension (LOH) | 1.33 (0.87-2.04; 0.179) | 1.18 (0.74-1.87; 0.467) | 0.98 (0.60-1.58; 0.935) |
| Glucose-related hypertension (GRH) | 3.49 (1.83-6.65; <0.001) | **3.45 (1.80-6.61; <0.001)** | 2.03 (0.83-4.98; 0.118) |
| Lipid-related hypertension (LRH) | 1.19 (0.76-1.88; 0.430) | 1.01 (0.62-1.63; 0.953) | 1.02 (0.61-1.72; 0.913) |
|  | **Outcome: cardiovascular mortality** | | |
|  | n=3,327; events=247 | n=3,312; events=247 | n=3,312; events=247 |
| Early-onset hypertension (EOH) | 1 | 1 | 1 |
| Late-onset hypertension (LOH) | 1.16 (0.57-2.34; 0.666) | 1.04 (0.49-2.21; 0.910) | 0.79 (0.35-1.76; 0.571) |
| Glucose-related hypertension (GRH) | **5.52 (2.34-13.01; <0.001)** | **5.40 (2.18-13.37; <0.001)** | 1.68 (0.50-5.63; 0.369) |
| Lipid-related hypertension (LRH) | 1.04 (0.53-2.04; 0.892) | 0.93 (0.46-1.87; 0.850) | 0.90 (0.44-1.87; 0.795) |

Model 1 reports the association of the outcome and phenotype membership after adjusting for sex and age. Model 2 additionally included race, current smoking status, and self-reported history of heart attack and stroke. Results in bold are statistically significant. These regression models accounted for the complex survey design of NHANES.
